# Coordination of microbe–host homeostasis by crosstalk with plant innate immunity

**DOI:** 10.1038/s41477-021-00920-2

**Published:** 2021-05-24

**Authors:** Ka-Wai Ma, Yulong Niu, Yong Jia, Jana Ordon, Charles Copeland, Aurélia Emonet, Niko Geldner, Rui Guan, Sara Christina Stolze, Hirofumi Nakagami, Ruben Garrido-Oter, Paul Schulze-Lefert

**Affiliations:** 1 Department of Plant Microbe Interactions, Max Planck Institute for Plant Breeding Research, Cologne, Germany; 2 Jiangsu Key Laboratory for Microbes and Functional Genomics, Jiangsu Engineering and Technology Research Center for Industrialization of Microbial Resources, College of Life Sciences, Nanjing Normal University, Nanjing, China; 3 Department of Plant Molecular Biology, Biophore, UNIL-Sorge, University of Lausanne, Lausanne, Switzerland; 4 Protein Mass Spectrometry Group, Max Planck Institute for Plant Breeding Research, Cologne, Germany; 5 Cluster of Excellence on Plant Sciences (CEPLAS), Max Planck Institute for Plant Breeding Research, Cologne, Germany

**Keywords:** Plant immunity, Microbiology

## Abstract

Plants grown in natural soil are colonized by phylogenetically structured communities of microbes known as the microbiota. Individual microbes can activate microbe-associated molecular pattern (MAMP)-triggered immunity (MTI), which limits pathogen proliferation but curtails plant growth, a phenomenon known as the growth–defence trade-off. Here, we report that, in monoassociations, 41% (62 out of 151) of taxonomically diverse root bacterial commensals suppress *Arabidopsis thaliana* root growth inhibition (RGI) triggered by immune-stimulating MAMPs or damage-associated molecular patterns. Amplicon sequencing of bacterial 16S rRNA genes reveals that immune activation alters the profile of synthetic communities (SynComs) comprising RGI-non-suppressive strains, whereas the presence of RGI-suppressive strains attenuates this effect. Root colonization by SynComs with different complexities and RGI-suppressive activities alters the expression of 174 core host genes, with functions related to root development and nutrient transport. Furthermore, RGI-suppressive SynComs specifically downregulate a subset of immune-related genes. Precolonization of plants with RGI-suppressive SynComs, or mutation of one commensal-downregulated transcription factor, *MYB15*, renders the plants more susceptible to opportunistic *Pseudomonas* pathogens. Our results suggest that RGI-non-suppressive and RGI-suppressive root commensals modulate host susceptibility to pathogens by either eliciting or dampening MTI responses, respectively. This interplay buffers the plant immune system against pathogen perturbation and defence-associated growth inhibition, ultimately leading to commensal–host homeostasis.

## Abstract (web summary)

Plants evolved powerful mechanisms to fight against pathogenic microorganisms. So how can they accept and even favour the presence of growth-promoting fungi or bacteria? Here, the authors show that helpful commensal bacteria can suppress part of the plant innate immune system.

## Main

Ubiquitous interactions within and between microbial communities and their plant hosts often shape host phenotypes and drive community diversification, leading to the conceptualization of plants and their associated microbes as discrete ecological units, or holobionts^1^. Analysis of *A. thaliana* grown in different locations has shown that plants accommodate a conserved core microbiota—microbial assemblages that represent a subset of microbes from the surrounding soil seeding inocula^2–5^. Although most microbiota members are commensals, a small number provide beneficial services for the host^6,7^, or become pathogenic under favourable conditions. Recent studies have shed light on how specialized metabolites^8–11^ and abiotic stresses^12,13^ influence host-associated microbiota. However, how microbe–host homeostasis is maintained after perturbation remains poorly understood.

Plants have evolved a sophisticated innate immune system to protect themselves against pathogens. One arm of this system is activated by the extracellular perception of microbe-associated molecular patterns (MAMPs)/PAMPs that are recognized by host pattern recognition receptors (PRRs). For example, the bacterial flagellin-derived epitope flg22 is detected by the cognate PRR FLS2. Both pathogenic and beneficial bacteria can carry flg22 epitope variants^14^, resulting in MTI^15,16^. MTI effectively restricts pathogen proliferation^17^ but, if unrestrained, MTI may result in plant growth penalties, a phenomenon known as the growth–defence trade-off^18^. Pathogens have evolved diverse mechanisms to suppress MTI^19^; however, this property is not limited to harmful bacteria, as a previous report has shown that commensal Alphaproteobacteria from the *Arabidopsis* root culture collection (*At*-RSPHERE)^20^ can also override flg22-mediated RGI^21^. Similarly, the beneficial rhizobacterium *Pseudomonas simiae* suppresses more than half of the MAMP-triggered transcriptional responses in monoassociation with *Arabidopsis*, possibly through acidification of the rhizosphere^14,22^. However, how plants tolerate a rich diversity of commensals without compromising effective resistance to pathogens is unknown. In this Article, we used a bottom-up approach to show that phylogenetically diverse root commensals can modulate plant immunity, and that their combined interactions in community contexts coordinate commensal–host homeostasis under pathogen challenges^23,24^.

## Results

### Taxonomically widespread ability of root commensals to interfere with defence-associated growth inhibition

**Fig. 1:**
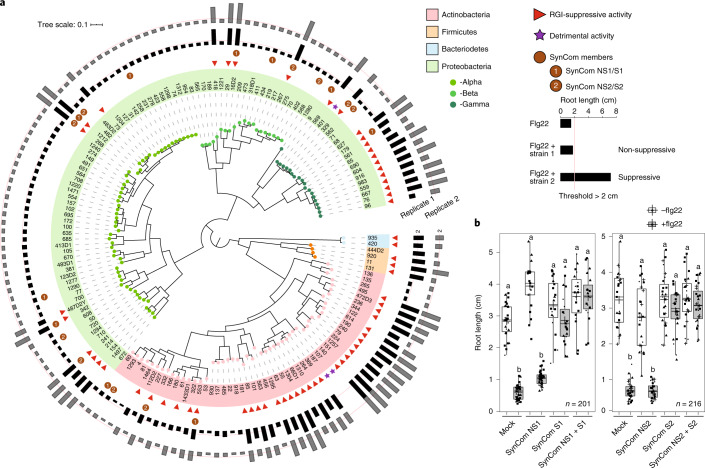
*At*-RSPHERE root commensals exhibit strain-specific variations to suppress flg22-mediated RGI in *pWER*::*FLS2-GFP* plants. **a**, Phylogenetic tree showing the distribution of strains exhibiting RGI-suppressive activity. The outer rings represent root lengths of plants (aged 3 weeks) germinated on plates supplemented with 1 μM flg22 and individual strains (OD_600_ = 0.0005). *n* = 2 independent replicates. The threshold for suppressive activity is indicated by the red line, that is, root length > 2 cm. **b**, The impact of four independent five-member SynComs (Supplementary Table 1), which differed in RGI-suppressive activity, on flg22-mediated RGI. Plants (aged 2 weeks) were germinated on plates supplemented with 1 μM flg22 and the indicated SynCom. Shapes represent three (SynCom NS1 + S1) and four (SynCom NS2 + S2) independent replicates in **b**. The *n* values indicate the total number of biological samples. Different letters indicate statistical significance, determined using two-sided Dunn’s Kruskal–Wallis test (*P* < 0.05). The box plots centre on the median and extend to the 25th and 75th percentiles, and the whiskers extend to the furthest point within 1.5× the interquartile range. Source data

**Extended Data Fig. 1:**
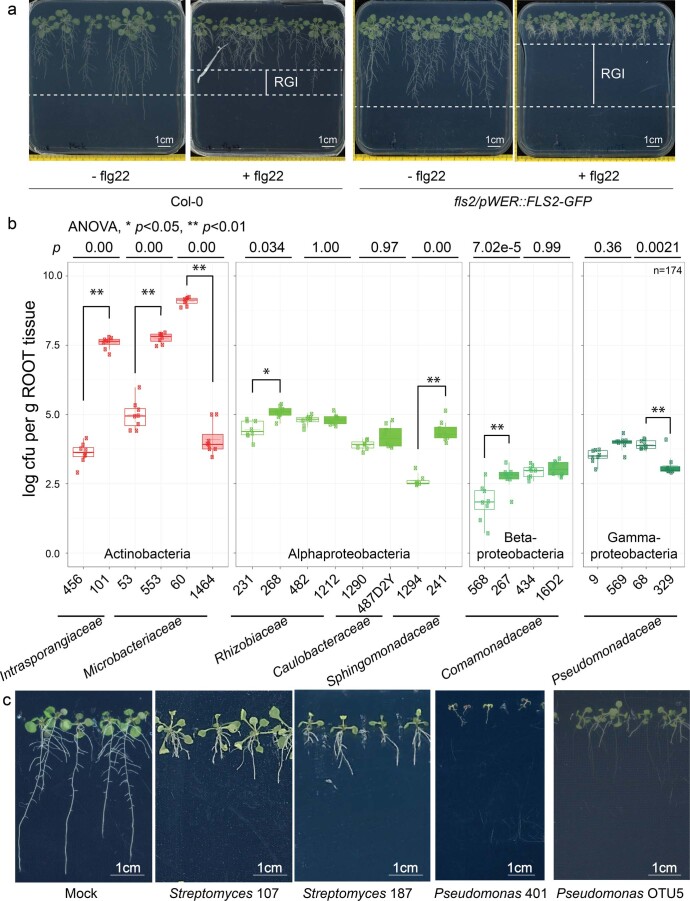
*pWER::FLS2-GFP* transgenic Arabidopsis plants are hypersensitive to flg22. **a**, Representative images of 3-week-old plants treated with 1 μM flg22 grown on agar plate. **b**, Bacteria load on 3-week-old WT roots inoculated with the indicated commensal. Boxes with filling background represent strains with RGI suppressive activity and colors correspond to phylogeny. n=total number of biological samples collected from one replicate. Asterisks indicate statistical significance (two-sided ANOVA, *p* < 0.05). The box plots centre on the median and extend to the 25th and 75th percentiles, and the whiskers extend to the furthest point within the range of 1.5× the interquartile range. **c**, Representative images of 2-week-old Col-0 germinated with the indicated strains with detrimental impact. Source data

To facilitate the screening of individual root commensals of the *At-*RSPHERE culture collection, we took advantage of a flg22-hypersensitive line, *pWER*::*FLS2-GFP*^25,26^, in which the flg22 receptor *FLS2* is overexpressed but restricted to the root epidermis. This hypersensitivity leads to an enhanced signal-to-noise ratio for flg22-mediated RGI (Extended Data Fig. 1a). After three weeks of co-culturing with individual bacterial isolates and flg22, 41% of the strains (62 out of 151) were found to interfere with RGI. RGI-suppressive activity was detected across all four phyla of the microbiota—Actinobacteria, Proteobacteria, Bacteroidetes and Firmicutes—but was overrepresented among Actinobacteria and Gammaproteobacteria commensals (Fig. 1a). Viable plate counting confirmed that the RGI-non-suppressive strains still colonize roots in mono-associations (Extended Data Fig. 1b). By contrast, only three strains, *Streptomyces* strains 107 and 187 and *Pseudomonas* 401, had detrimental impacts on *Arabidopsis* in monoassociations; *Pseudomonas* 401 most severely compromised plant growth (Extended Data Fig. 1c).

**Extended Data Fig. 2:**
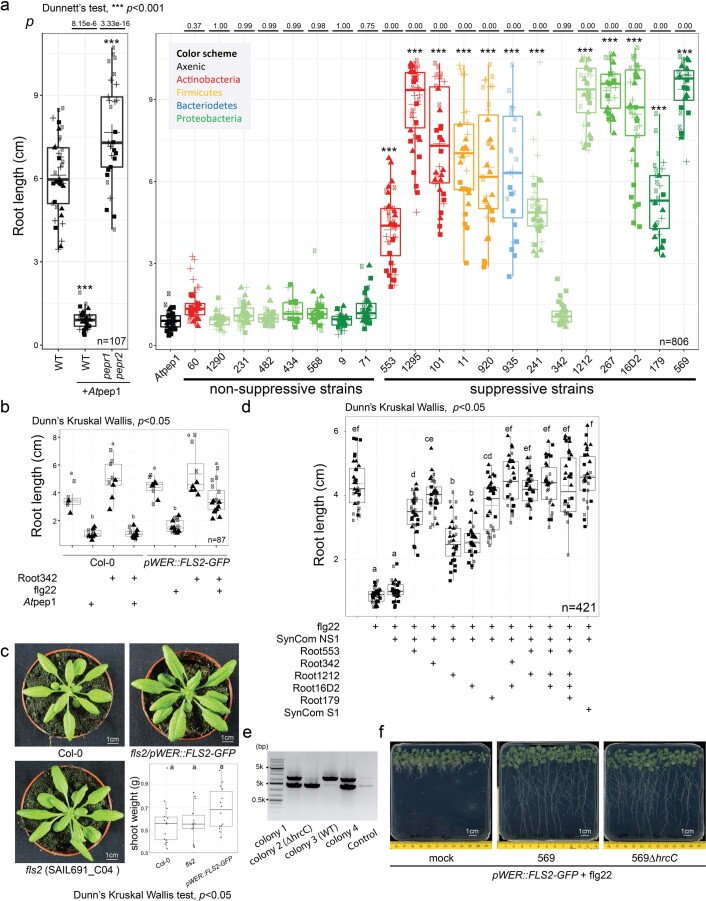
Microbiota members modulate root growth inhibition (RGI) induced by *At*pep1 and function quantitatively in community contexts. **a**, Root lengths of WT and *pepr1pepr2* mutants (left panel) or WT plants (right panel) inoculated with 1 μM *At*pep1 alone or with individual strain. **b**, *Caulobacter* 342 exhibits differential ability to suppress flg22- or *At*pep1-mediated RGI. **c**, Representative images of WT, *fls2* and *pWER::FLS2-GFP* plants grown in natural Lausanne soil and their corresponding shoot fresh weight without any flg22 treatment. **d**, Root lengths of *pWER::FLS2-GFP* plants inoculated with different combination of non-suppressive and suppressive strains in the presence of 1 μM flg22. n=total number of biological samples collected from four (**a**), two (**b**), two (**c**) and three (**d**) biological replicates. Asterisks indicate statistical significance (two-sided Dunn’s Kruskal Wallis or two-sided Dunnett’s test, *p* < 0.05). The box plots centre on the median and extend to the 25th and 75th percentiles, and the whiskers extend to the furthest point within the range of 1.5× the interquartile range. **e**, Gel blot (Source ED Fig. 2e) showing the result of PCR genotyping of *569*Δ*hrcC* generated by homologous recombination. Lane from left to right, GeneRuler 1 kb plus ladder, four individual colonies and control. **f**, Root lengths of *pWER::FLS2-GFP* plants coinoculated with flg22 and 569 or *569*Δ*hrcC*. Source data

To examine whether root-derived bacteria were also able to suppress RGI elicited by an endogenous plant-derived danger-associated molecular pattern (DAMP), we treated plants with the DAMP *At*pep1, which induces RGI and immune responses^27^. Using *At*pep1-treated Col-0 wild type (WT) plants, we found that 12 out of 13 suppressive strains, representing members from diverse taxa, retained the ability to interfere with RGI, whereas none of the eight non-suppressive strains elicited this effect (Extended Data Fig. 2a). Thus, phylogenetically diverse root commensals can suppress both DAMP- and MAMP-induced RGI. One isolate, *Caulobacter* strain 342, suppressed flg22- but not *At*pep1-mediated RGI (Extended Data Fig. 2b), suggesting the existence of at least two modes of RGI suppression: one interfering with both MAMP- and DAMP-induced RGI, and the other possibly specific to flg22 perception.

Although germ-free *pWER*::*FLS2-GFP*^25^ plants respond to flg22 treatment with enhanced RGI compared with Col-0 on synthetic medium, no growth differences were noted between these two genotypes when grown on natural soil (Extended Data Fig. 2c). Given that root growth in natural soil probably proceeds in the face of chronic exposure to MAMPs and DAMPs, as well as colonization by both suppressive and non-suppressive commensals, we speculated that the aforementioned RGI suppression phenotype may act as a dominant community trait. To test this hypothesis, we composed four independent but taxonomically similar five-member SynComs with contrasting abilities for RGI suppression, that is, non-suppressive SynComs (SynCom NS1 and NS2) and suppressive SynComs (SynCom S1 and S2; Supplementary Table 1). We observed RGI-suppressive activity in plants inoculated with the suppressive SynComs, but not in plants inoculated with the non-suppressive SynComs. Furthermore, full RGI-suppressive activity was retained when these commensals were combined as ten-member SynComs (Fig. 1b). A recent study showed that auxin-mediated RGI could be rescued by *Variovorax* commensals^28^. However, our four tested SynComs did not induce RGI to a level comparable to flg22 treatment, and the presence of *Variovorax* 434 in SynCom NS1 did not rescue the flg22-mediated RGI phenotype (Fig. 1b). Thus, we conclude that RGI is mainly caused by flg22 treatment, and is widely suppressed by *At*-RSPHERE members that function dominantly in our set-up.

We speculated that the co-occurrence of RGI-non-suppressive and suppressive strains might reflect a need for commensal microbes to dampen plant immunity to balance root growth and defence trade-offs. We therefore examined whether a single suppressive strain is sufficient to achieve full RGI suppression. We found that the addition of diverse individual suppressive strains to a five-member non-suppressive SynCom resulted in only partial RGI suppression (Extended Data Fig. 2d). This result suggests that the identity of suppressive commensals, as well as the input proportion of suppressive to non-suppressive strains, affect RGI-suppression capacity quantitatively.

**Extended Data Fig. 3:**
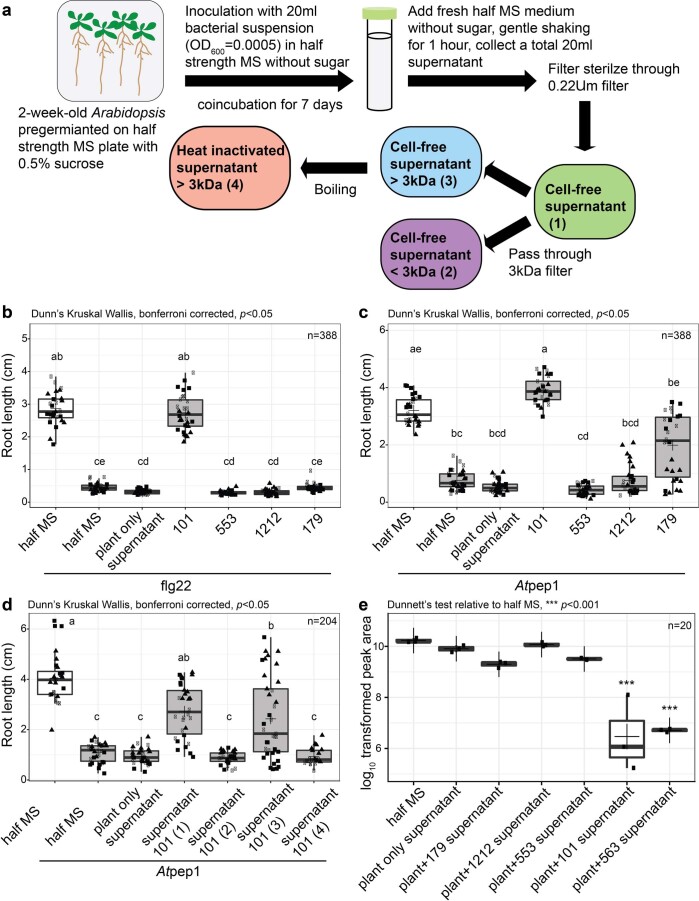
Commensals can degrade/modify flg22 *in vitro*. **a**, Schematic diagram showing how bacterial supernatants are collected. Root lengths of plants coinoculated with the supernatant of different bacteria and **b**, 1 μM flg22 (*pWER::FLS2-GFP*) or **c**, 1 μM *At*pep1 (Col-0). All strains used in these experiments exhibited RGI suppressive activity as shown in Fig. 1. **d**, Root lengths of Col-0 plants treated with the supernatant of Janibacter 101 after different treatments shown in (**a**). **e**, Mass spectrometry analyses detecting the intact flg22 peptide (QRLSTGSRINSAKDDAAGLQIA [M + 4H] 568.8056) by estimating the peak area generated by Skyline. All strains used in this experiment exhibited suppressive activity on flg22-mediated RGI. n=total number of biological samples collected from three biological replicates (ED Fig. 3b–e). Asterisks or different letters indicate statistical significance (two-sided Dunn’s Kruskal Wallis or two-sided Dunnett’s test, *p* < 0.05). The box plots centre on the median and extend to the 25th and 75th percentiles, and the whiskers extend to the furthest point within the range of 1.5× the interquartile range. Source data

The ability of specific strains to differentially suppress *At*pep1- and flg22-mediated RGI prompted us to investigate the mechanisms that underlie this biological process. Previously, commensal *Pseudomonas* spp. in monoassociations were shown to acidify the growth medium, rendering plants insensitive to flg22 (ref. ^22^). To determine whether acidification is responsible for RGI suppression by our SynCom, we measured the growth medium pH of plants that were co-inoculated with different SynComs. We observed average reductions in pH, ranging from pH 5.18 in mock treated plants to pH 4.62 and pH 3.97 in the presence of a SynCom S1 and NS1, respectively. This lack of correlation between RGI suppression and growth medium acidification suggests that this mechanism is unlikely to explain suppression in our community set-up. We next investigated whether type-III secretion, which is a well-characterized virulence mechanism among Gram-negative bacteria pathogens, is required for the suppressive activity of root commensals tested. Interestingly, *hrcC*—a gene that is essential for a functional type-III secretion system in pathogenic *Pseudomonas*—is dispensable for RGI suppression mediated by suppressive *Pseudomonas* strain 569 (Extended Data Fig. 2e,f). We next investigated whether root commensals can target the step upstream of flg22 perception. Only the culture filtrate of *Janibacter* 101, an Actinobacteria member, but not that of three other suppressive strains, derepressed both flg22 and *At*pep1-induced RGI (Extended Data Fig. 3a–c). Heat treatment and filtration of the culture filtrate showed that the molecule(s) responsible for the suppressive activity retained in the supernatant of *Janibacter* 101 is heat-labile, and is larger than 3 kDa (Extended Data Fig. 3d). Mass spectrometry analysis further revealed that the filtrates of *Janibacter* 101 and the closely related suppressive *Janibacter* 563, but not three other tested suppressive commensals, elicited a significant reduction in intact flg22 peptide (Extended Data Fig. 3e). Thus, the ability of these two *Janibacter* strains to suppress MTI resembles the activity of pathogenic bacteria^29^, and is associated with an extracellular mechanism that can modify/degrade flg22 peptide. Together, these data reveal that commensals use diverse mechanisms to suppress elicitor-mediated RGI.

### Activation of immunity shapes root microbiota establishment

**Fig. 2:**
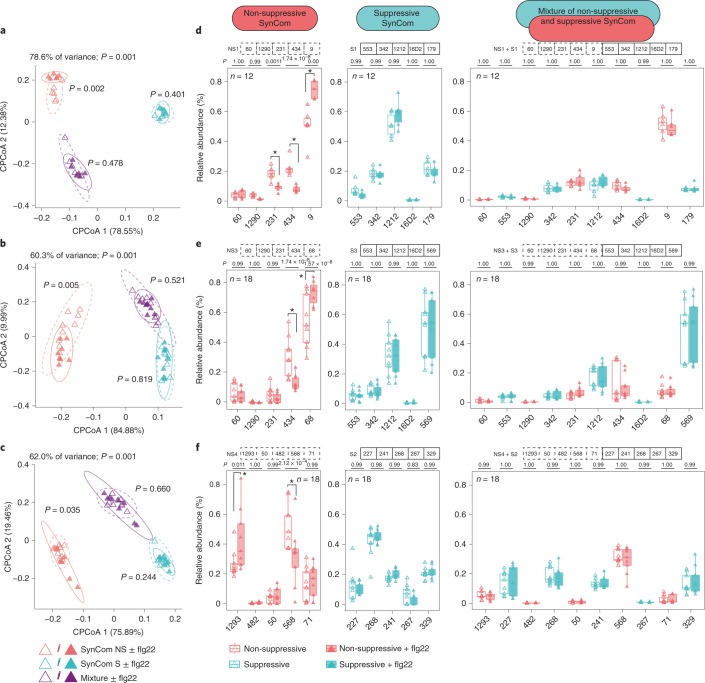
Activation of immunity by flg22 affects community establishment. **a**–**c**, Constrained coordination of the microbial profile of *pWER*::*FLS2-GFP* root samples showing the corresponding community shift of non-suppressive SynCom after treatment with flg22. Ellipses correspond to Gaussian distributions fitted to each cluster (95% confidence interval). *P* values next to the ellipses indicate statistical significance determined using a permutational analysis of variance (PERMANOVA) test between untreated and flg22-treated samples of each SynCom (permutation = 999, *P* < 0.05). **d**–**f**, The RA of strains after treatment with flg22. Three experiments were performed: experiment 1 (**a** and **d**); experiment 2 (**b** and **e**); and experiment 3 (**c** and **f**). These experiments were conducted using different SynComs and were repeated twice with consistent results. The corresponding strains used in each SynCom are indicated at the top of **d**–**f**. Non-suppressive and suppressive strains are indicated by the dashed and solid lines, respectively. The values in parentheses are eigenvalues explained by the principal component. The colours indicate the SynComs used and the shapes indicate flg22 treatment. *n* values indicate the total number of biological samples collected from three independent replicates. Asterisks (*) indicate statistical significance determined using two-sided ANOVA (*P* < 0.05) and the *P* values are provided at the top of each graph. The box plots centre on the median and extend to the 25th and 75th percentiles, and the whiskers extend to the furthest point within 1.5× the interquartile range.

**Extended Data Fig. 4:**
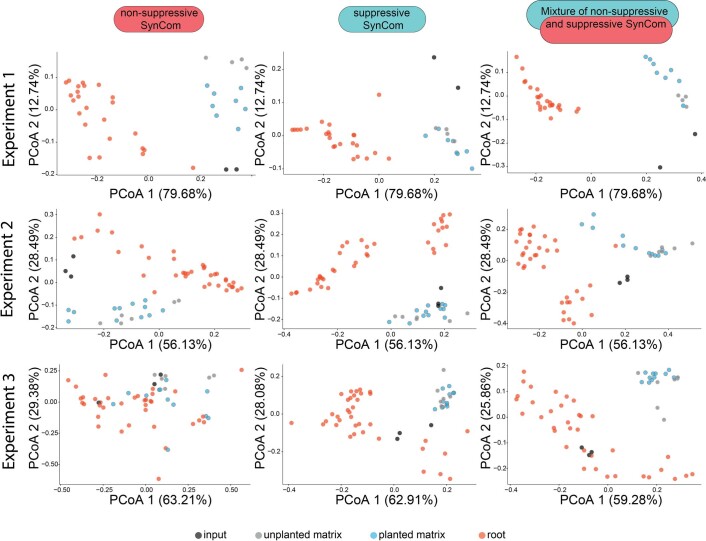
Compartment specific effect on community establishment. Unconstrained PCA plots showing the compartment-specific effect of bacteria community two weeks after inoculation on agar plates. The root samples include both Col-0 and *pWER::FLS2-GFP* plants.

**Extended Data Fig. 5:**
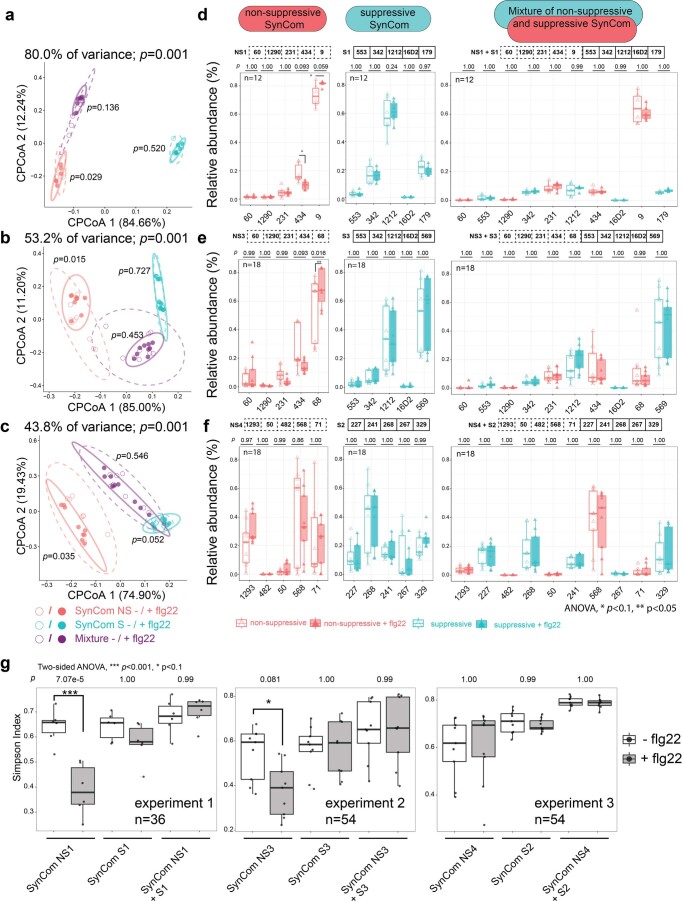
Activation of immunity by flg22 affects community establishment in WT Col-0 plants. **a-c**, Constrained coordination of the microbial profile of Col-0 root samples showing the effect of flg22 and SynCom. Ellipses correspond to Gaussian distributions fitted to each cluster (95% confidence interval). *p*-values next to ellipses indicate statistical significance based on a PERMANOVA test between untreated and flg22-treated samples of each SynCom (permutation = 999, *p* < 0.05). **d-f**, Relative abundance of strains upon flg22 treatment. Experiment 1: (**a**,**d**); experiment 2: (**b**,**e**); experiment 3: (**c**,**f**). The corresponding strains used in each SynCom were indicated on top of (**d**-**f**). These experiments were conducted using different SynComs and repeated twice with consistent result. Values in bracket are eigenvalues explained by the Principal Component (PC). Colors indicate SynComs used and shapes indicate flg22 treatment. **g**, Simpson diversity index of the corresponding experiments performed with *pWER::FLS2-GFP* plants. n=total number of biological samples collected from three biological replicates. Asterisks indicate statistical significance (two-sided ANOVA, *p* < 0.05) and the *p* values are provided on top of each graph. The box plots centre on the median and extend to the 25th and 75th percentiles, and the whiskers extend to the furthest point within the range of 1.5× the interquartile range.

To determine whether plant immunity affects microbiota establishment, we performed reconstitution experiments with gnotobiotic plants grown on an agar matrix. We designed three taxonomically similar SynComs with contrasting RGI suppression abilities for community profiling experiments at a strain-specific resolution (a total of six SynComs; the SynComs used in experiment 1 and 2 differ in two Gammaproteobacteria, and the SynComs used in experiment 3 are composed of entirely different strains; Supplementary Table 1). Principal coordinate analyses (PCoA) of Bray–Curtis dissimilarities revealed that root-associated bacterial communities were distinct from the corresponding unplanted or planted matrix samples (Extended Data Fig. 4), regardless of the SynCom composition and plant genotypes (Col-0 and *pWER*::*FLS2-GFP*). Constrained PCoA revealed that flg22 treatment elicited a consistent community shift in plants inoculated with non-suppressive SynComs, while samples from those inoculated with suppressive SynComs remained together. Consistent with a dominant effect of RGI suppression, roots inoculated with ten-member mixed communities (suppressive plus non-suppressive SynComs) were not affected by flg22 treatment (Fig. 2a–c and Extended Data Fig. 5a–c). This is consistent with another report showing that roots of *fls2* mutant and Col-0 plants have similar community profiles, consisting of a mixed 32-member SynCom^30^.

To dissect the contribution of individual strains to the overall community shift, we quantified the relative abundance (RA) of individual strains. The detection of non-suppressive commensals as the most abundant strains in the mixed SynComs suggests that the ability to dominate in a community is not necessarily coupled to RGI suppression (Fig. 2d–f and Extended Data Fig. 5d–f). However, the RA of specific strains in a community was impacted by plant immunity. For example, flg22 treatment led to an altered RA of *Pseudomonas* 9/68 (increased) and *Variovorax* 434 (decreased), while Microbacteriaceae 60 was unaltered (experiments 1 and 2). Similarly, flg22 treatment altered the RA of Microbacteriaceae 1293 (increased) and Comamonadaceae 568 (decreased), while *Pseudomonas* 71 was unaffected (experiment 3; Fig. 2d–f). A similar trend was also detected in Col-0 (Extended Data Fig. 5d–f), although the effect was more pronounced in *pWER*::*FLS2-GFP* plants, possibly due to enhanced MTI and/or altered root architecture. Furthermore, we found that flg22 treatment reduced within-sample diversity of non-suppressive SynComs (experiments 1 and 2; Extended Data Fig. 5g), suggesting that immune activation can affect the distribution of specific strains in community contexts.

### Root transcriptomic changes and dampening of immunity by suppressive SynComs

**Fig. 3:**
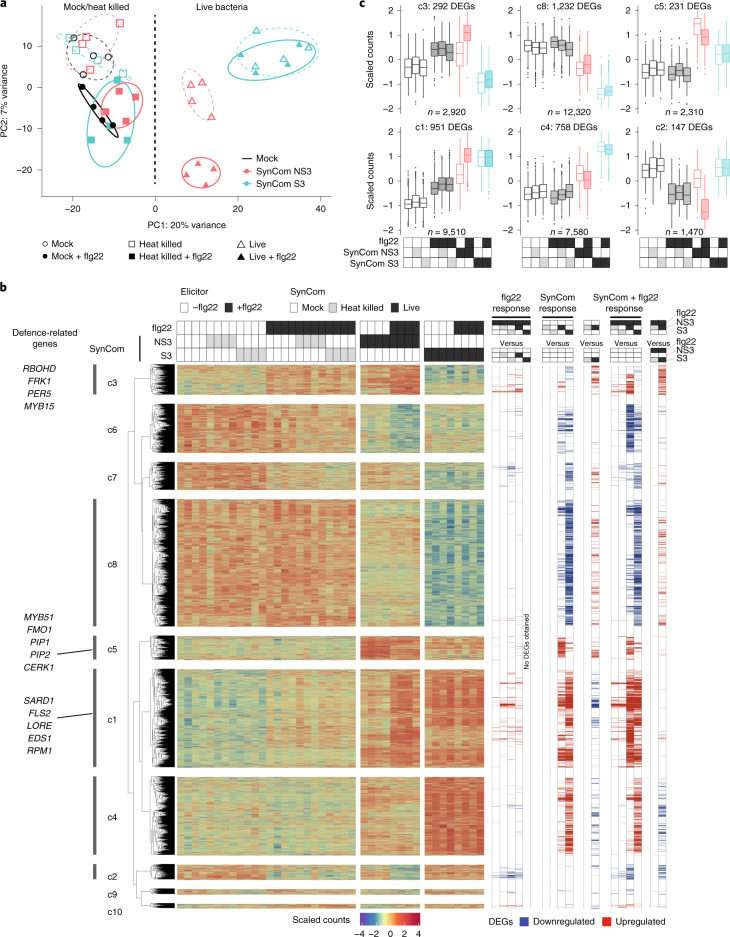
SynCom colonization and flg22 treatment induce root transcriptomic changes in WT Col-0 plants. **a**, PCA plot separating samples inoculated with SynComs and flg22. Ellipses correspond to *t*-distributions fitted to each cluster (70% confidence interval). **b**, Heat map (middle) and DEGs (Supplementary Tables 5–7) obtained by pairwise comparison (right). *k*-means clusters (*k* = 10) are marked on the left. **c**, Scaled counts of transcripts in six clusters and their expression patterns after treatments. *n* values indicate the total number of biological samples collected from four replicates. The corresponding transcriptome data of *pWER*::*FLS2-GFP* plants are presented in Extended Data Fig. 6 and Supplementary Tables 2–4. The colours used in **a** and **c** match those used in Fig. 2, and correspond to different SynComs. The box plots centre on the median and extend to the 25th and 75th percentiles, and the whiskers extend to the furthest point within 1.5× the interquartile range.

**Extended Data Fig. 6:**
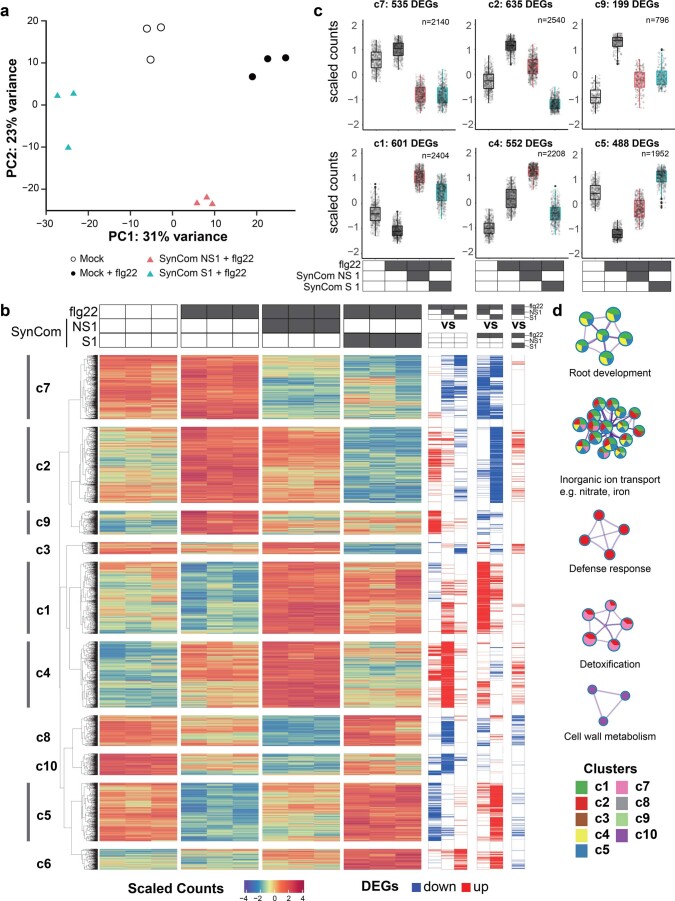
SynCom colonization and flg22 treatment induce global root transcriptomic changes of *pWER::FLS2-GFP* plants. **a**, PCA plot separating samples inoculated with taxonomically similar SynComs (SynCom NS1 and S1) treated with 1 µM flg22. **b**, Heat map (middle) and DEGs (Supplementary Table 2–4) obtained by pairwise comparison under flg22 treatment (right). *k*-means clusters (*k* = 10) with hieratical tree are marked on the left. **c**, Scaled counts of transcripts in six clusters and their expression patterns upon treatments. n=total number of biological samples collected from three replicates. The box plots centre on the median and extend to the 25th and 75th percentiles, and the whiskers extend to the furthest point within the range of 1.5× the interquartile range. **d**, A subset of significantly changed GO terms (adjusted *p* < 0.05) associated with specific clusters. Size of pie charts corresponds to number of DEGs. Linked GO terms indicate shared genes (Jaccard similarity > 0.2).

Although flg22-mediated RGI is closely associated with immune activation, its role as a bona fide immune output is unclear. Here, we sought to explore how inoculation with suppressive or non-suppressive SynComs affected the root transcriptome of plants treated with flg22 and grown on an agar matrix (Supplementary Tables 2–7). Principal component analyses (PCA) at the transcriptome level revealed distinct expression patterns between Col-0 plants inoculated with live bacteria, compared with germ-free plants (PC1, 20% variance; Fig. 3a). Interestingly, the transcriptional output of roots inoculated with these two taxonomically similar SynComs was clearly distinguishable after two weeks of co-cultivation, even in the absence of flg22 treatment (Fig. 3a, triangles). Furthermore, we observed a separation according to the immune status of the plants, triggered by flg22 exposure, in all of the samples treated with heat-killed bacteria as well as with the non-suppressive SynCom (PC2, 7% variance; Fig. 3a). By contrast, flg22 treatment of plants colonized by the suppressive SynCom did not elicit significant changes. Independent transcriptome experiments using *pWER*::*FLS2-GFP* plants confirmed these results (Extended Data Fig. 6 and Supplementary Tables 2–4).

**Extended Data Fig. 7:**
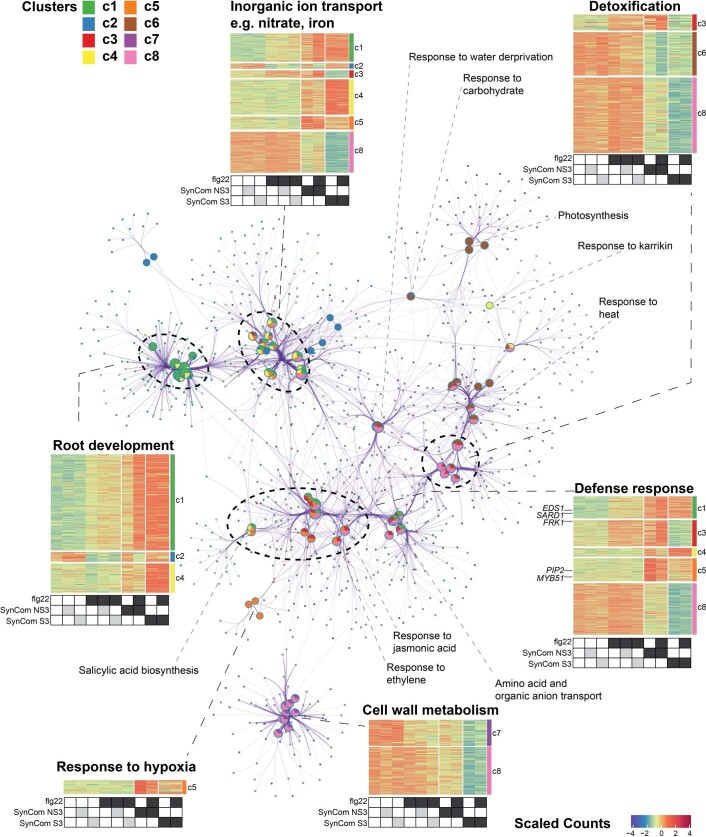
DEG-linked GO network. Network showing the association of significantly changed biological function GO terms (pie charts; adjusted *p* < 0.05) with different DEGs (small nodes) obtained by pairwise comparison in Col-0 transcriptome experiments. Size of pie charts corresponds to number of DEGs. Linked GO terms indicate shared genes (Jaccard similarity > 0.2). Closely gathered GO terms form clusters in the network showing similar functions (indicated with dash lines), and heatmaps for GO clusters corresponding to the average scaled counts of transcripts for each treatment are drawn on the sides. The position of clusters is arranged according to topology. Clusters and DEGs correspond to Fig. 3b.

**Extended Data Fig. 8:**
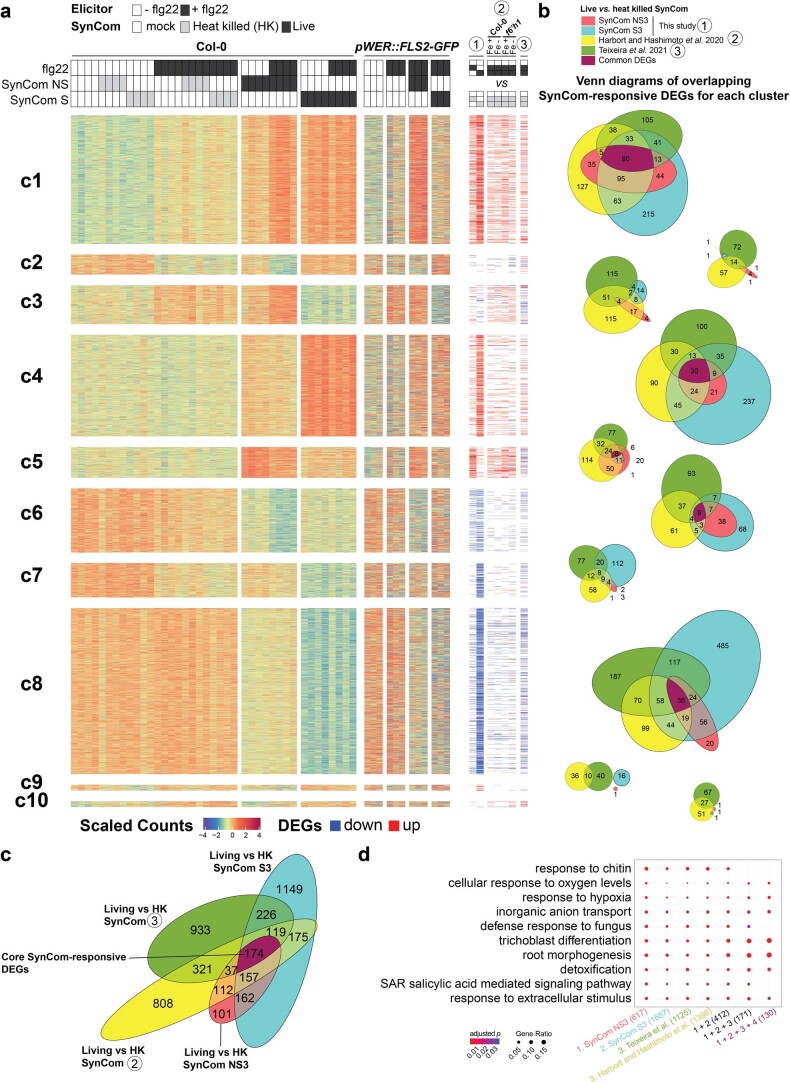
Live SynComs induce common host transcriptomic response. **a**, Heat map summarizes the root transcriptomic changes from three independent studies (Supplementary Table 8–12, ①: current study; ②: Harbort and Hashimoto et al., 2020 (ref. ^13^); ③: Teixeira et al., 2021 (ref. ^31^)). The data obtained from *pWER::FLS2-GFP* plants was shown alongside Col-0 dataset indicating conserved trends of gene expression across different genotypes. DEGs were obtained by pairwise comparisons between live and heat-killed (HK) SynCom treatments (adjusted *p*-value < 0.05 and fold-change > 1.5). The corresponding data extracted from Harbort and Hashimoto et al., 2020 (ref. ^13^) and Teixeira et al., 2021 (ref. ^31^) were shown on the right. **b**, Venn diagrams showing the overlapping SynCom-responsive DEGs (live *vs*. HK SynComs) of each cluster. Most SynCom-responsive DEGs were found in c1, c4, c5, c6 and c8. **c**, Venn diagram summarizing the overlap of SynCom-responsive DEGs of these independent studies. The core 174 SynCom-responsive host DEGs were highlighted in purple. **d**, Enrichment of selected GO terms for the 174 core SynCom-responsive DEGs. Numbers in parentheses indicate the total number of DEGs with GO annotations.

Next, we performed *k*-means clustering of differentially expressed genes (DEGs) involved in the flg22 response, the SynCom response or both (Fig. 3b and Supplementary Tables 5–7). We observed three large clusters (2,221 DEGs) that were induced (c4 and c5) or suppressed (c8) by live bacteria, independent of flg22 treatment (Fig. 3b,c). Gene Ontology (GO) enrichment analyses showed that the SynCom-responsive clusters were primarily enriched in functions related to detoxification, root development, nutrient transport and response to hypoxia (Extended Data Fig. 7). To determine whether similar GO terms could also be identified in experiments with more complex SynComs, we compared our data with two independent *Arabidopsis* root transcriptome studies that used SynComs consisting of both suppressive and non-suppressive commensals (35 members^31^ and 115 members^13^). Despite differences in technical set-ups and SynCom complexities, we identified 174 common SynCom-responsive DEGs in the absence of flg22 that were related to the same biological functions mentioned above (Extended Data Fig. 8 and Supplementary Tables 8–12).

Importantly, we found a flg22-inducible cluster (c3), which was significantly upregulated by the non-suppressive SynCom, but downregulated by the suppressive community (Fig. 3b,c), in a pattern matching the RGI phenotype of the plant (Fig. 1b) and the bacterial community shifts (Fig. 2). As expected, a portion of defence-related genes were enriched in c3, such as *PER5*, *FRK1* and *RBOHD* (70 genes; Fig. 3b). However, additional defence-related DEGs were found outside c3 and were upregulated by flg22 treatment, even in the presence of the suppressive SynCom (348 genes; Fig. 3b). Previously characterized examples include regulators of antimicrobial camalexin, for example, *MYB51* (refs. ^32,33^) (c5); systemic acquired resistance, for example, *FMO1* (c5) and *SARD1* (c1)^34,35^; and endogenous peptides amplifying MTI, for example, *PIP1* and *PIP2* (ref. ^36^) (c5; Fig. 3b).

**Extended Data Fig. 9:**
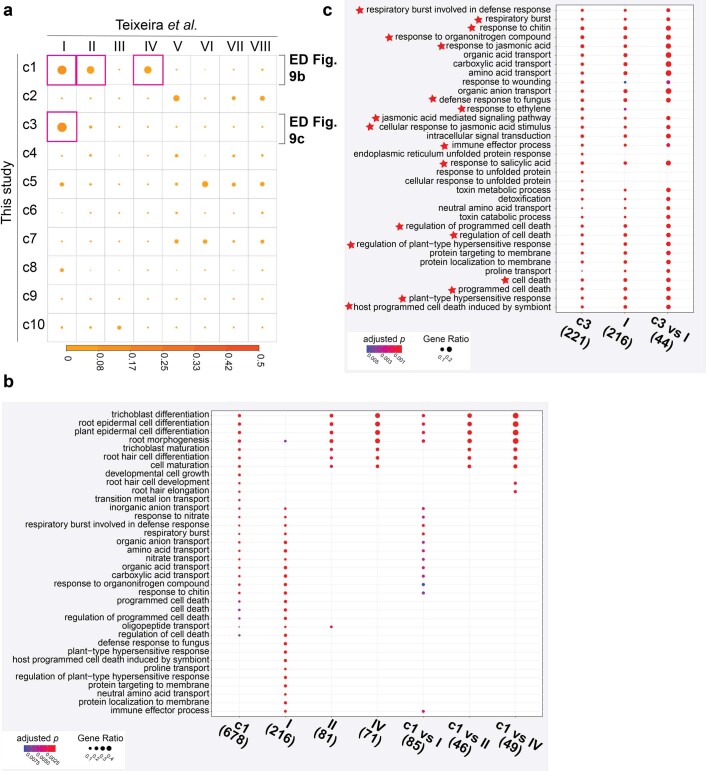
A subset of flg22-induced genes is downregulated by SynComs. **a**, Pairwise comparison of Jaccard similarity between clusters from two root transcriptome studies (this study: 10 clusters by row; and 8 clusters by column: Teixeira et al., 2021 (ref. ^31^)). Significantly enriched GO terms associated with cluster 1 (**b**; root development related) and cluster 3 (**c**; defense related) showing highest similarity between studies were shown. 58 common DEGs were downregulated by SynComs across these two studies, and 21 out of 44 GO annotated DEGs shown in (**c**) were defense-related. Defense-related GOs were highlighted by a star. Numbers in parentheses indicate the total number of DEGs with GO annotations.

Recent research showed that MAMP responsiveness in germ-free roots was gated by the expression of damage-induced PRRs^37^. However, the sustained expression of *FLS2* (c1) in the presence of SynComs indicates that RGI suppression is not due to *FLS2* downregulation (Fig. 3b). The ability of diverse root commensals to suppress *At*pep1-mediated RGI (Extended Data Fig. 2a) also highlights the interference from *FLS2*-independent pathway(s). An independent study by Teixeira et al. also identified a cluster of DEGs that was highly induced in axenic *Arabidopsis* by treatment with flg22, but suppressed by the presence of a 35-member SynCom consisting of suppressive and non-suppressive root commensals^31^ (Extended Data Fig. 9). Remarkably, this cluster showed the largest overlap with our cluster c3, with 58 common DEGs (at least 21 were defence-related) that were downregulated by both SynComs (Extended Data Fig. 9). Even though we have shown that two *Janibacter* strains can degrade/modify flg22 extracellularly, the downregulation of only a subset of flg22-mediated responses suggests that the direct removal of the flg22 peptide is insufficient to account for the differential suppressive activities observed.

**Extended Data Fig. 10:**
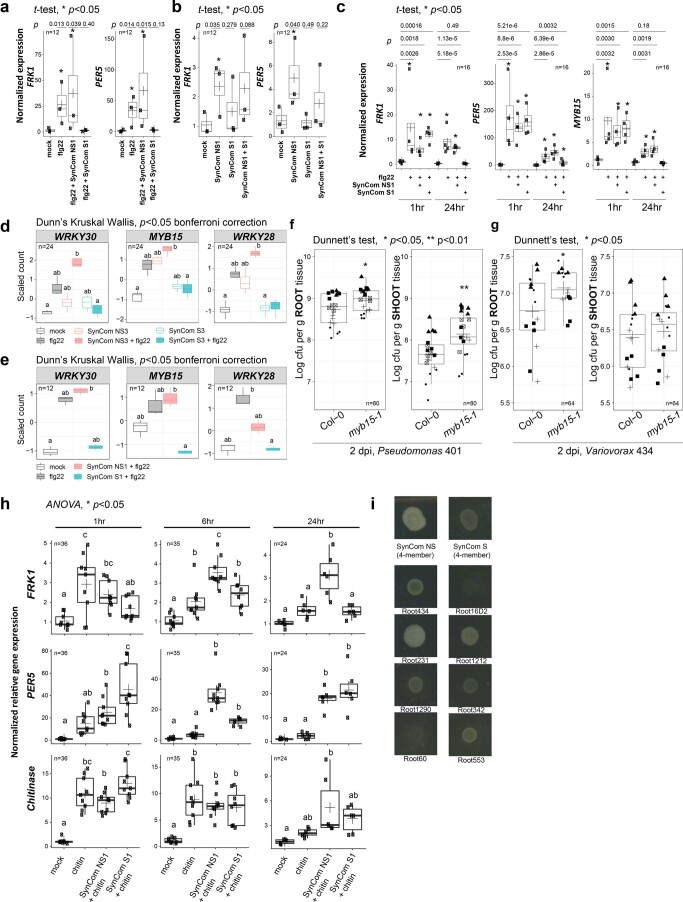
*MYB15* is a positive regulator of defense against specific commensals. **a**,**b**, Expression of flg22-induced marker genes, *PER5* and *FRK1*, in roots of 14-day-old *pWER::FLS2-GFP* plants upon treatment with the indicated SynCom. **c**, Expression of *FRK1*, *MYB15* and *PER5* of Col-0 plants 1 hr and 24 hr post flg22 treatment. Plants were precolonized by the indicated SynCom for two weeks. Expression patterns of *WRKY28*, *WRKY30* and *MYB15* in roots of 14-day-old (**d**) Col-0 and (**e**) *pWER::FLS2-GFP* transcriptome experiments. *myb15-1* mutant enhances proliferation of *Pseudomonas* 401 (**f**) and *Variovorax* 434 (**g**). **h**, Expression of *FRK1*, *PER5* and chitinase (*AT2G43620*) upon coinoculation between chitin (1 mg/ml) and SynComs at the indicated time points. Plants were precolonized by the indicated SynCom for two weeks. **i**, Individual strains or combined SynComs do not antagonize 401 growth *in vitro*. Individual strain was spotted on a background lawn of 401 and halo-of-inhibition (if any) was noted 5 dpi. n=total number of biological samples collected from three (**a**-**b**, **g**, 1 and 6 hr), four (**c**) or two biological replicates (**g**, 24 hr). Asterisks and different letters indicate statistical significance. One-sided student’s *t*-test (**a**-**c**), two-sided Dunn’s Kruskal Wallis (**d**-**e**), two-sided Dunnett’s test (**f**-**g**) and two-sided ANOVA (**h**) were used, *p* < 0.05). The box plots centre on the median and extend to the 25th and 75th percentiles, and the whiskers extend to the furthest point within the range of 1.5× the interquartile range. Source data

We further validated our findings by examining the expression of two flg22-inducible defence marker genes^12,25,38^ in roots of *Arabidopsis* using quantitative PCR (qPCR) in the presence of other suppressive SynComs. *PER5* and *FRK1* remained significantly elevated two weeks after co-inoculation with flg22 and a non-suppressive SynCom, but not with a suppressive SynCom (Extended Data Fig. 10a). A non-suppressive SynCom alone also significantly induced the expression of *PER5* and *FRK1*, indicating that non-suppressive commensals stimulate specific root immune responses. As expected, a ten-member mixed SynCom, which was shown to suppress flg22-mediated RGI (Fig. 1b), did not significantly induce the expression of *PER5* and *FRK1* (Extended Data Fig. 10b). We next examined whether the suppressive SynCom exerted an effect on the root defence response at earlier time points after flg22 stimulation. Intriguingly, we observed a significant induction of *PER5*, *FRK1* and *MYB15* 1 h after flg22 treatment. However, suppressive SynCom, in contrast to non-suppressive SynCom, specifically downregulated the expression of these three genes after 24 h (Extended Data Fig. 10c), suggesting that SynComs can modulate defence responses as early as 1 d after stimulation.

To determine whether MTI has a direct impact on commensal proliferation independent of any microbe–microbe interactions, we focused on transcription factors (TFs) and investigated the contributions of the top three candidates identified in our dataset—*WRKY30*, *MYB15* and *WRKY28* (cluster c3; Extended Data Fig. 10d,e). Null mutants of *WRKY30* and *WRKY28* have not been reported, and our attempts to knock out these TFs using CRISPR failed, suggesting that these genes are essential for plant viability^39,40^. We therefore focused on *MYB15*, a positive regulator of defence against the foliar pathogen *Pto*DC3000 (ref. ^41^). In *myb15-1* plants, elimination of this single TF led to a significantly enhanced proliferation of the detrimental strain *Pseudomonas* 401, and the commensal *Variovorax* 434 (*P* < 0.05; Extended Data Fig. 10f,g), which also showed a reduced RA after flg22 treatment in community contexts (Fig. 2d,e). Together, amplicon sequencing and transcriptome data support the idea that colonization of specific root commensals is affected by host MTI, which can be attenuated by suppressive strains.

Next, we tested whether our suppressive SynCom can also suppress defence responses triggered by a non-proteinaceous elicitor, chitin, which is commonly found as a MAMP in the cell wall of eukaryotic fungi. We performed a time-resolved experiment to follow the expression of the defence marker genes *FRK1*, *PER5* and chitinase (*AT2G43620*) 1 h, 6 h and 24 h after elicitor application. In contrast to flg22, chitin treatment only marginally induced *FRK1* expression after 1 h, whereas *PER5* and *AT2G43620* were significantly induced up to 6 h (Extended Data Fig. 10h). This is consistent with a previous report showing that flg22 and chitin induce both overlapping and specific root responses^14^. No stimulation of marker gene expression was detected after 24 h chitin application. Interestingly, our SynComs exerted a cooperative effect on chitin-mediated signalling. For example, chitin-induced *PER5* expression was further stimulated by a suppressive SynCom after 1 h but this stimulation was reversed such that the non-suppressive SynCom enhanced *PER5* expression after 6 h. After 24 h, both suppressive and non-suppressive SynComs induced *PER5* expression, while the chitin-triggered response was no longer detectable (Extended Data Fig. 10h), suggesting that our SynComs interact differently with flg22- and chitin-triggered responses.

### Suppressive and non-suppressive commensals differentially impact plant susceptibility to opportunistic pathogens

**Fig. 4:**
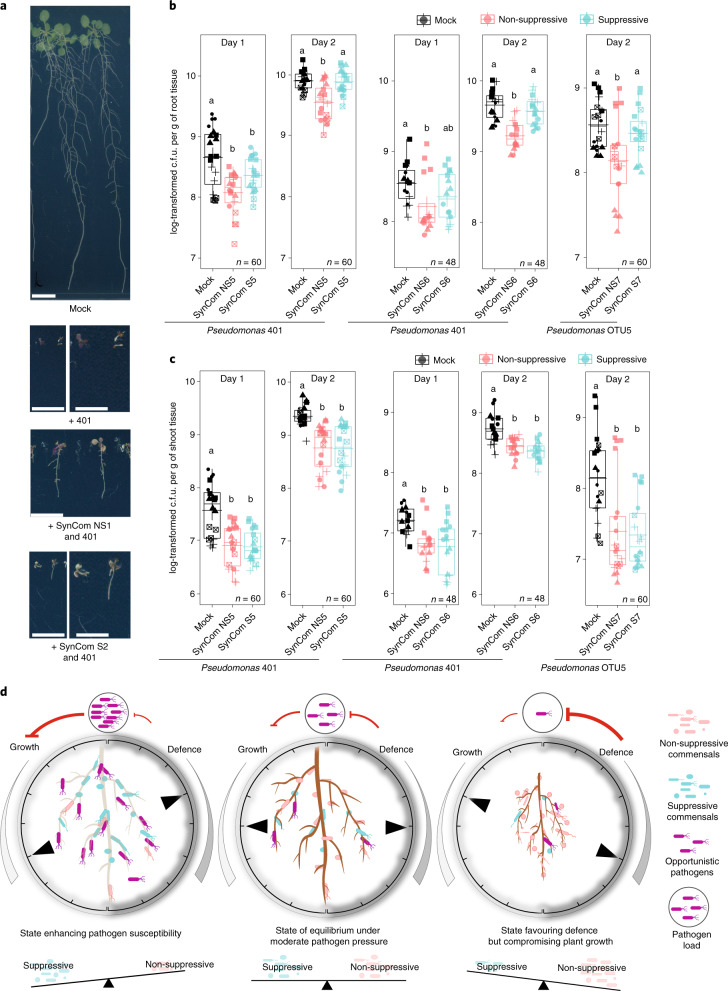
Imbalance of specific bacteria impacts plant susceptibility to opportunistic *Pseudomonas* pathogens. **a**, Symptoms of WT plants (aged 3 weeks) germinated with the indicated SynCom and *Pseudomonas* 401. Scale bars, 1 cm (top and third row, and second and fourth row (left)) and 0.5 cm (second and fourth row (right)). **b**,**c**, Bacterial titre of *Pseudomonas* 401 and OTU5 on the roots (**b**) and shoots (**c**) of *pWER*::*FLS2-GFP* plants that were precolonized with the indicated SynComs for 2 weeks. No flg22 was used in these experiments. The shapes represent five (SynCom NS5 + S5), four (NS6 + S6) and five (NS7 + S7) biological replicates in **b**. c.f.u., colony-forming units. *n* values indicate the total number of biological samples. Different letters indicate statistical significance determined using two-sided Dunn’s Kruskal–Wallis test (*P* < 0.05). The box plots centre on the median and extend to the 25th and 75th percentiles, and the whiskers extend to the furthest point within 1.5× the interquartile range. **d**, The ‘rheostat model’ proposes that the balance between non-suppressive and suppressive strains integrates with plant innate immunity, and buffers the system against pathogen challenge and defence-associated trade-off. Source data

As a subset of commensals dampens root immune responses, we hypothesized that colonization with a suppressive SynCom might render plants more susceptible to opportunistic pathogens. We identified three detrimental strains from *At*-RSPHERE. In particular, *Arabidopsis* plants inoculated with *Pseudomonas* 401 exhibited reduced growth and accumulated pigments in shoots reminiscent of stress-inducible anthocyanins (Extended Data Fig. 1c), which indicates its pathogenic potential in a laboratory environment. Consistent with the fact that *Pseudomonas* 401 was originally isolated from healthy and asymptomatic *Arabidopsis* roots colonized by a diverse microbial community, the detrimental effect was attenuated when plants were colonized by our SynComs. Interestingly, the attenuation was stronger when plants were co-colonized with the non-suppressive SynCom, compared with the suppressive SynCom (Fig. 4a).

Recent reports suggest that there is a positive correlation between disease progression in natural *Arabidopsis* populations and bacterial biomass^42,43^. To determine whether the virulence of *Pseudomonas* 401 is related to enhanced plant colonization, we quantified its absolute abundance on *pWER*::*FLS2-GFP* precolonized with suppressive or non-suppressive SynComs. Plants already colonized by suppressive SynComs harboured significantly higher *Pseudomonas* 401 titres compared with plants that were precolonized with non-suppressive SynComs (Fig. 4b,c). Interestingly, this SynCom-dependent difference seemed to be limited to roots, as *Pseudomonas* 401 growth in shoots was similarly restricted by co-colonization with either community (Fig. 4b,c). Even though we are not excluding an impact of microbe–microbe interactions through niche competition, none of the individual strains antagonized *Pseudomonas* 401 in vitro (Extended Data Fig. 10i). These data further suggest that the underlying growth differences are probably not the result of antibiosis.

To determine whether SynComs modulate plant susceptibility to a characterized opportunistic pathogen prevalent in natural *A. thaliana* populations, and exclude the possibility that differential impact of root commensals in roots and shoots is a result due to niche specialization of *Pseudomonas* 401 on roots only, we inoculated plants with the opportunistic *Pseudomonas* leaf pathogen OTU5 (isolate p5.e6) (Extended Data Fig. 1c). Plants colonized by suppressive SynComs supported higher growth of *Pseudomonas* OTU5 compared with plants colonized by non-suppressive SynComs, and this SynCom-specific effect was again observed only in roots and not in shoots (Fig. 4b,c). Together with the RNA-sequencing (RNA-seq) and targeted PCR data, these results suggest that precolonization with non-suppressive SynComs activated root immunity and this correlates with reduced growth of the tested opportunistic pathogens, whereas suppressive SynComs failed to provide pathogen protection.

## Discussion

In nature, a subset of soil-dwelling bacteria colonizes roots seemingly without influencing host traits, and are therefore often considered to be commensals. Here, using a bottom-up approach, we show that phylogenetically diverse commensals, representing the core of the *Arabidopsis* root microbiota^20^, share the ability to suppress host defence responses, a microbial trait that is dominant in our community set-up and is therefore easily overlooked in nature. The functional redundancy of members of the root microbiota to interfere with the host’s immune response is consistent with our observation that mixed communities consisting of non-suppressive and suppressive strains resist flg22-mediated community changes otherwise detected in non-suppressive SynCom-colonized plants (Fig. 2). This finding might explain why immune-related *Arabidopsis* mutants, tested in natural soil, show relatively mild changes in root microbiota composition^10^. Here we showed that the reduction in apoplastic pH and potential type-III secretion system-dependent mechanisms are insufficient to fully account for our MTI-suppression phenotype. Two closely related *Janibacter* strains, but not three other tested suppressive commensals, were shown to degrade/modify flg22. Even though we cannot rule out the possible involvement of specific plant metabolites produced after interactions with the suppressive bacteria, our findings suggest that root commensals can use multiple mechanisms to target host immune responses, rendering this community trait difficult to overcome by the plant host.

To date, information on *Arabidopsis* root transcriptomic changes evoked by commensals are limited to monoassociations^14,21^, leaving a gap in our understanding of how plant roots respond to commensal communities that can reach a steady-state as early as 13 d after inoculation^44^. We unexpectedly found that, after 2 weeks, root colonization by taxonomically similar commensal SynComs, differing in their ability to suppress RGI, elicited 2,221 DEGs (clusters c4, c5, c8) with remarkable overall similarity. These changes, which are associated with presumably steady-state SynComs, contrast with the subtle response to heat-killed SynComs or flg22 treatment alone, reflecting an impact of active commensal colonization on host transcriptional outputs beyond plant responses to chronic exposure to MAMPs. Furthermore, we observed robust enrichment of specific GO terms related to root development, nutrient transport, response to hypoxia and detoxification across experimental set-ups and SynCom complexities^13,31^). Indeed, rhizobacteria alone are known to modulate root traits^21,45,46^. In return, root-secreted photoassimilates feed up to 20% of root-associated bacteria^47^. As photoassimilates have been shown to serve as sources of organic carbon that limit bacterial growth^48^, we speculate that the enrichment of these GOs is associated with altered nutrient flux, and reduced oxygen due to microbial respiration in roots. Although our SynComs are taxonomically diverse with predicted varied metabolic repertoires^20,49^, convergence to core transcriptomic outputs indicate integrated responses to a state of ‘community commensalism’.

The zigzag model of the plant immune system proposes that effective resistance is the result of quantitative outputs above a certain threshold following MAMP perception^19^. Colonization by suppressive SynComs led to the downregulation of a subset of flg22-induced genes (Fig. 3, cluster c3), whereas colonization by non-suppressive SynComs alone stimulated these genes and further upregulated their expression together with flg22. Thus, the responsiveness of these defence-associated genes to SynCom colonization differs greatly with respect to the ability of the bacterial community to suppress RGI. However, roots in nature are co-colonized by both groups of commensals, and our experiments indicate a quantitative output that is dependent on their ratio. Intriguingly, recent studies reported that 42% (ref. ^22^) and 28% (ref. ^31^) of commensals from two other *Arabidopsis* root-derived culture collections quench early and late flg22-induced responses in monoassociations, respectively^22^. Together with our study, this confirms the potential of the root microbiota to modulate plant growth–defence traits.

We hypothesize that the imbalance between non-suppressive and suppressive commensals might reduce plant fitness under stress conditions. Indeed, plants that are precolonized by suppressive SynComs are as susceptible as germ-free plants to opportunistic *Pseudomonas* pathogens, whereas plants associated with non-suppressive SynComs are more resistant, but prone to MAMP-induced RGI. The observed defence-associated community shifts and potentially reduced alpha-diversity might hinder the provision of microbiota-derived beneficial services^50^, or exert a detrimental impact on the host under dysbiosis^51^. Thus, we propose a rheostat model (Fig. 4d) in which a balance between commensals with contrasting MTI-modulating activities constitutes an integral feature of the holobiont to buffer plant resistance to pathogen perturbation and defence-associated growth reduction.

It remains unclear which factors govern the state of equilibrium and the corresponding ratio between non-suppressive and suppressive strains. However, on the basis of the results obtained using three pairs of SynComs (Fig. 2), the initial input composition has a key role in defining the equilibrium state. The timing of colonization^44^ and abiotic factors^52^ probably also exert an influence. As a consequence, these complex interactions enable community coexistence, and ultimately establish microbe–host homeostasis. Accordingly, their ratio will impact the amplitude and/or might set the threshold for effective resistance in the zigzag model. Plants in nature are influenced by fluctuating stresses and are colonized by more diverse microbial communities that modulate plant physiology through multiple mechanisms, including the modulation of phytohormone signalling^28,53^. As our SynComs are constructed on the basis of their ability to suppress flg22-mediated responses, we found that they exert a synergistic effect on chitin-mediated responses. Selective modulation of chitin and flg22-mediated responses—for example, by the cytoplasmic receptor-like kinase PBL27—has been reported^54^. The characterization of the molecular mechanisms that underlie immunosuppression by root commensals may identify immunity components that are specific to one or integrate multiple upstream signalling pathways. Another future task will be to test whether the rheostat model also applies to communities with different traits to alleviate abiotic stresses.

## Methods

*Arabidopsis thaliana* ecotype Columbia (Col-0, CS60000) and *pepr1pepr2* were laboratory stocks. *myb15* (ref. ^41^) (SALK_151976) was a gift from N. Clay (Yale University, USA). The transgenic line *pWER*::*FLS2-GFP*^25^ (*fls2*: SAIL691_C04 background) was provided by N. Geldner (Université de Lausanne, Switzerland). flg22 (QRLSTGSRINSAKDDAAGLQIA) and *At*pep1 (ATKVKAKQRGKEKVSSGRPGQHN) peptides were synthesized by EZbiolab. Chitin was purchased from Sigma-Aldrich (C9752).

### Growth conditions for plants

*Arabidopsis* seeds were surface-sterilized in 70% ethanol twice for 5 min each followed by a brief wash with 100% ethanol. Seeds were then washed three times with sterile water. Cold-stratified seeds were sowed on agar plates (1%, Difco Agar Granulated, BD Biosciences, discontinued) or Bacto agar (BD Biosciences) prepared with half-strength Murashige and Skoog (MS) medium (Duchefa) and 0.1 g l^−1^ 2-(*N*-morpholino)ethanesulfonic acid (pH 5.7). Sugars were not provided as an additional carbon source unless otherwise specified. Plants were grown under short-day conditions (10 h light–14 h dark) under a 21 °C–19 °C cycle, 65% relative humidity and a light intensity of 120 mE m^−2^ s^−1^. For experiments involving *myb15-1*, surface-sterilized seeds were sowed on half-strength MS agar plates supplemented with 5 g l^−1^ sucrose.

### Culture conditions for bacteria

Information on individual strains used can be found at *At*-RSPHERE (http://www.at-sphere.com/)^20^. OTU5 (p5.e6)^42^ was provided by D. Weigel (Max Planck Institute for Developmental Biology, Tübingen, Germany). Bacterial strains were prepared by taking an aliquot from the glycerol stock, followed by incubation on 50% tryptic soy broth (TSB) agar plates (Sigma-Aldrich) at 25 °C for 1–4 d. Before the start of the experiments, strains were cultured in 50% TSB medium to saturation, and subcultured to log phase with fresh medium at a 1:5 ratio. Bacterial cultures were pelleted by centrifugation at 8,000*g* for 5 min, followed by two washes with 10 mM MgSO_4_.

### Screening for RGI-suppressive strains in monoassociation

After washing, bacteria were diluted with 10 mM MgSO_4_ to an optical density at 600 nm (OD_600_) concentration of about 0.1. A total of 150 µl bacterial suspension was added to still warm 50 ml half-strength MS agar medium at a final bacterial concentration of OD_600_ = 0.0005. A final concentration of 1 µM flg22 was added accordingly. Plates were dried for 2 h before approximately 15 surface-sterilized *pWER*::*FLS2-GFP* seeds were sowed on each plate. The expression of the flg22 receptor FLS2 in *pWER*::*FLS2-GFP* is limited to the root epidermis such that potential interorgan shoot-to-root signal after flg22 perception is minimized. The plates were sealed with 3M tape and transferred to the phytochamber for incubation. One week after germination, plants with delayed germination were removed and the plates were trimmed to about ten remaining plants. Pictures were taken 3 weeks after incubation and the primary root lengths were quantified using ImageJ. Shoots were separated from the roots and the fresh shoot weight of individual plants was taken. For experiments using 1 µM *At*pep1, wild-type Col-0 plants were used instead.

A phylogenetic tree of selected strains from *At*-RSPHERE was performed previously^20^ and visualized using iTOL^55^. Strains leading to a rescue of RGI with a root length longer than 2 cm (average root length of germ-free flg22-treated *pWER*::*FLS2-GFP* plants = 1.53 cm; *n* = 37) after coinoculation with 1 µM flg22, and exhibiting consistent suppressive activity across two biological replicates, were considered to be suppressive. Suppressive strains are indicated by a red triangle in Fig. 1a. For the inoculation of SynCom, each bacterium was inoculated to a final concentration of OD_600_ = 0.0005, that is, for a five-member SynCom, the total bacteria added was OD_600_ = 0.0025. The five-member SynCom is composed of Actinobacteria, Alpha-, Beta- and Gammaproteobacteria. Bacteroidetes and Firmicutes were not included in these SynComs as no strains with differential ability to suppress RGI were identified in these two phyla. The composition of SynComs used in this manuscript can be found in Supplementary Table 1.

### 16S amplicon sequencing and community profiling

For 16S community profiling, root samples were harvested and libraries were processed according to previously a published protocol^4^. In brief, plants were germinated with the indicated SynCom in the presence or absence of 1 µM flg22, and incubated for 14 d before harvesting. Plants were inoculated with SynCom NS1 and S1 for experiment 1 (Fig. 2a,d and Extended Data Fig. 5a,d); SynCom NS3 and S3 for experiment 2 (Fig. 2b,e and Extended Data Fig. 5b,e); and SynCom NS4 and S2 for experiment 3 (Fig. 2c,f and Extended Data Fig. 5c,f). Plant roots were separated from the shoots and pooled from three plates from each biological replicate. Roots were washed briefly with sterile water and blotted dry before being transferred into Lysing Matrix E tubes (MP Biomedicals) at −80 °C until processing. Samples were homogenized using a Precellys 24 homogenizer (6,200 r.p.m. twice for 30 s with 15 s pauses in between; Bertin Technologies). Total root and bacteria DNA was extracted using the FastDNA SPIN Kit for Soil (MP Biomedicals) according to the manufacturer’s instructions, eluted in 80 μl elution buffer and quantified using the Quant-iT PicoGreen dsDNA Assay (Thermo Fisher Scientific). Samples were diluted to 3.5 ng μl^−1^, and 3 µl samples were used in a three-step PCR amplification protocol as follows.

Step 1: the V5V7 region of the bacterial 16S rRNA gene was amplified in triplicate reactions using the primers 799F and 1192R in a 25 µl reaction volume containing 2 U DFS-Taq DNA polymerase (Bioron), 1× incomplete buffer, 2 mM MgCl_2_, 0.3% bovine serum albumin, 0.2 mM dNTPs (Life Technologies) and 0.3 μM forward and reverse primers. The same PCR parameters were used for each primer pair (initial denaturation at 94 °C for 2 min, denaturation at 94 °C for 30 s, annealing at 55 °C for 30 s, extension at 72 °C for 30 s, repeat steps 2–4 for 25 cycles, and final extension at 72 °C for 10 min). Primers and proteins were digested by adding 1 μl of Antarctic phosphatase, 1 μl exonuclease I and 2.44 μl Antarctic phosphatase buffer (New England Biolabs) to 20 μl of the pooled replicate reactions at 37 °C for 30 min, followed by enzyme deactivation at 85 °C for 15 min. Reactions were centrifuged for 15 min at 4,000 r.p.m. and 3 μl of supernatant was used for the second PCR step in triplicate reactions.

Step 2: PCR reactions were performed as stated above with the number of cycles reduced to ten using primer pairs 799F and individual reverse barcoded primers. PCR quality and quantity were estimated by loading 5 μl of each reaction on a 1.5% agarose gel. Approximately similar amounts of DNA sample from the same biological replicate were pooled, and the mixtures were loaded onto a 1.5% agarose gel. DNA bands with the correct size were cut out and purified using the QIAquick Gel Extraction Kit (QIAGEN).

Step 3: Gel-purified DNA was used as a template for the third PCR using forward barcoded primers and p7_pad_R with a total of ten cycles. PCR reactions were loaded onto a 1.5% agarose gel and DNA bands with the correct size were cut out and purified using the QIAquick Gel Extraction Kit (QIAGEN). Double-barcoded DNA was purified and concentrated using Agencourt AMPure XP beads. The concentration of the purified DNA was determined using Quant-iT PicoGreen dsDNA Assay (Thermo Fisher Scientific). Paired-end Illumina sequencing was performed using 20 ng µl^−1^ of the final library in-house, using the MiSeq sequencer and custom sequencing primers.

### Amplicon data analysis

Forward and reverse sequencing reads were denoised and demultiplexed separately according to the barcode sequence using QIIME^56^ using the following parameters: phred = 30; bc_err = 2. After quality-filtering and merging the paired-end reads, amplicon tags were next aligned to a reference set of sequences obtained from the whole-genome assemblies of every strain included in each experiment using USEARCH (uparse_ref command)^57^. A count feature table for each strain was generated using only perfect matches to the reference sequence from the genome collection. This count table was used for subsequent diversity and enrichment analyses. Alpha and beta diversities were calculated after normalizing count tables to the total number of reads per sample. The Simpson index was obtained using the diversity function in the vegan package. The Bray–Curtis dissimilarity index was calculated using the vegdist function in the vegan package^58^ and used for unconstrained ordination by PCoA. All data were used except for biological replicate c of experiment 1 due to a potential contamination issue or PCR error. Constrained PCoA was performed using the vegan capscale function on the Bray–Curtis dissimilarity matrices, constraining by the interaction between flg22 treatment and SynCom variables and conditioning by technical parameters. Statistical significance of separation between community profiles according to flg22 treatment was determined using PERMANOVA with 999 permutations (anova.cca function in vegan). Finally, all amplicon data were visualized using the ggplot2 (ref. ^59^) R package.

### Preparation of cell-free bacterial supernatant

Col-0 (15–20 plants) plants were pregerminated on half-strength MS agar plate supplemented with 5 g l^−1^ sucrose. After two weeks, plants were submerged in 20 ml washed bacterial suspension (OD_600_ = 0.0005) in half-strength MS medium without sucrose. After another 7 d of coincubation, fresh half-strength MS medium was added to obtain a final 20 ml supernatant after 1 h of gentle shaking. The supernatant was filter-sterilized by passing through 0.22 µm PES filter (Millipore). The filtrates were separated into two fractions by passing through the 3 kDa ultracentrifugal filter (4,000 r.p.m. for 60 min; Amicon Millipore). The filtrates were filter-sterilized by passing through the 0.22 µm filter again, if necessary. Finally, the fraction larger than 3 kDa was heat-inactivated by boiling for 5 min. To test for RGI-suppressive activity, surface-sterilized seeds were germinated in 1 ml supernatant, supplemented with 5 g l^−1^ sucrose and 1 µM *At*pep1 or flg22 in a 12-well plate for 2 weeks.

### Generation of the Δ*hrcC* mutant of *Pseudomonas* strain 569

The *Pseudomonas* 569 deletion mutant was generated using a homologous recombination protocol^60^. In brief, PCR fragments flanking the upstream and downstream region of the *hrcC* gene were PCR-amplified and cloned into *pK18mobsacB*. The FRT-flanked cassette from *pCPP5209* was inserted between the two hrcC fragments. The resultant *pK18mobsacB*::*ΔhrcC* construct was electroporated into log-phase-grown *Pseudomonas* 569, which was prewashed and resuspended in 0.3 M sucrose, at 2.5 kV and 150 Ω using the Biorad electroporator. The transformant was selected on half of a TSB plate supplemented with 25 ng µl^−1^ gentamycin. The double-crossover deletion mutant was further confirmed by colony PCR and Sanger sequencing.

### Mass spectrometry

For in vitro detection of flg22, 1 µM flg22 was co-incubated with 1 ml supernatant for 1 h at room temperature. Half-strength MS medium without sugar was used as a control. Sample aliquots (100 µl) were mixed with 200 µl UA (8 M urea in 100 mM Tris-HCl pH 8.5) and adjusted to 10 mM dithiothreitol using 1 M stock. Samples were loaded onto 30 kDa spin filters (Vivacon 500, Sartorius) and centrifuged at 14,000*g* for 15 min. The filtrate was collected and loaded onto 2 kDa spin filters (Vivacon 500, Sartorius) and centrifuged at 14,000*g* for 30 min, after which 300 µl UA was added and the samples were centrifuged again (14,000*g* for 45 min, or until most liquid had passed through the filter). Next, 100 µl 55 mM chloroacetamide was added to the filter and samples were incubated for 30 min in the dark, after which they were centrifuged at 14,000*g* for 20 min. UA (300 µl) was added and the samples were centrifuged at 14,000*g* for 45 min. The samples were washed twice with 300 µl 100 mM Tris-HCl, pH 8.5, by centrifugation (14,000*g* for 45 min). For elution, 200 µl Tris-HCl was added, and the inverted spin filters were centrifuged at 2,000*g* for 2 min to collect eluate into a fresh tube. The eluates were desalted using StageTips with C18 Empore disk membranes (3M)^61^, and a final elution was performed using 40% acetonitrile and 0.1% trifluoroacetic acid. The samples were dried in a vacuum evaporator, and dissolved in 10 µl 2% acetonitrile (ACN) and 0.1% trifluoroacetic acid for analysis.

### Liquid chromatography coupled with tandem mass spectrometry data acquisition and data analysis

Samples were analysed using the EASY-nLC 1000 system (Thermo Fisher Scientific) coupled to a QExactive mass spectrometer (Thermo Fisher Scientific). Peptides were separated on 16 cm frit-less silica emitters (New Objective, 0.75 µm inner diameter), packed in-house with reversed-phase ReproSil-Pur C18 AQ 3 µm resin (Dr. Maisch). Peptides were loaded into the column and eluted for 50 min using a segmented linear gradient of 5% to 95% solvent B (0 min, 5% B; 0–5 min, 5% B; 5–25 min, to 20% B; 25–35 min, to 35% B; 35–40 min, to 95% B; 40–50 min, 95% B) (solvent A, 0% ACN and 0.1% formic acid; solvent B, 80% ACN and 0.1% formic acid) at a flow rate of 300 nl min^−1^. Mass spectra were acquired in data-dependent acquisition mode using a TOP10 method. Mass spectra were acquired in the Orbitrap analyzer with a mass range of 300–1,500 *m*/*z* at a resolution of 70,000 full width at half maximum, and a target value of 3 × 10^6^ ions. Precursors were selected with an isolation window of 2.0 *m*/*z*. HCD fragmentation was performed at a normalized collision energy of 25. MS/MS spectra were acquired with a target value of 5 × 10^5^ ions at a resolution of 17,500 full width at half maximum, a maximum injection time of 85 ms and a fixed first mass of 100 *m*/*z*. Peptides with a charge of 1, greater than 6 or with an unassigned charge state were excluded from fragmentation for MS^2^; dynamic exclusion for 20 s prevented repeated selection of precursors.

Raw data were directly analysed at the MS1 level using Skyline (https://skyline.ms)^62^, against the sequence of the flg22 peptide. LysC specificity was required, and a maximum of two missed cleavages was allowed. The minimum peptide length was set to 7 amino acids and the maximum length was set to 25 amino acids. Carbamidomethylation of cysteine, oxidation of methionine and protein N-terminal acetylation were set as modifications. The results were filtered for precursor charges of 2 and 3. Peaks of the intact flg22 peptide precursor were integrated manually, and peak areas were exported for further processing.

### Infection experiments

To detect any *Pseudomonas*-401-dependent symptoms and the corresponding attenuation by SynComs, plants were germinated with *Pseudomonas* 401 alone (OD_600_ = 0.0005; Extended Data Fig. 1c), or coinoculated with a SynCom for 2 weeks (Fig. 4a). For viable plate-counting experiments, plants were pregerminated on half-strength MS agar plates inoculated with the indicated strain or SynCom for 14 d (Fig. 4b,c). Plants were then flood inoculated with a bacterial suspension *of Pseudomonas* strain 401 or OTU5 (p5.e6) OD_600_ = 0.0001) in 10 mM MgSO_4_ supplemented with 0.005% silwet. Excessive liquid was removed 5 min after flood inoculation, and plants were transferred to a new half-strength MS agar for further incubation. After 1 d or 2 d, shoots and roots were separated, roots and shoots from five and three individual plants were pooled together after brief washing and blotted dry with sterile filter paper. The samples were homogenized with metal beads in 500 µl MgSO_4_ using a Precellys 24 homogenizer (6,200 r.p.m. twice for 30 s with 15 s pauses in between; Bertin Technologies). *Pseudomonas* strain 401 and OTU5 (p5.e6) were transformed with *pBBR1-MCS5* carrying a gentamycin-resistance cassette. Serial dilution was performed, and bacterial dilutions were spread on 50% TSB plates supplemented with 25 ng µl^−1^ gentamycin to select for the strain of interest until single colonies appeared.

### In vitro halo-of-inhibition assay

Washed *Pseudomonas* strain 401 (100 µl) was inoculated into 50 ml warm 25% TSB medium with an initial OD_600_ = 0.1. After solidification, 10 µl prewashed bacterial suspension prepared from 1 ml saturated overnight bacterial culture from individual strains was spotted onto the *Pseudomonas*-401-preinoculated plates. Any halo-of-inhibition was recorded up to 5 d after incubation at 25 °C.

### Transcriptome experiments

Plants were germinated with the indicated SynCom in the presence or absence of 1 µM flg22 and incubated for 14 d before harvesting. For transcriptome experiments, Col-0 plants were inoculated with SynCom NS3 and SynCom S3 (Fig. 3); *pWER*::*FLS2-GFP* plants were inoculated with SynCom NS1 and SynCom S1 (Extended Data Fig. 6). For transcriptome experiments, plants were not transferred to minimize induced damage. Roots from three plates (minimum of 15 plants) were combined as one replicate and a total of three replicates were sampled for each condition. Roots were washed briefly with sterile water and blotted dry before being transferred into Lysing Matrix E tubes (MP Biomedicals) at −80 °C until processing. Roots were homogenized with Lysing Matrix E using a prechilled adapter and the TissueLyser II (QIAGEN, 20 pulses per s for 1 min). RNA was extracted using the Plant RNeasy Mini Kit (QIAGEN) according to the manufacturer’s instructions. RNA quality was determined using a 2100 Bioanalyzer (Agilent Technologies). Preparation of Illumina sequencing libraries was conducted by the Max Planck Genome Center using an input of 1 μg total RNA. Sequences were generated using the Illumina HiSeq2500 platform. Approximately 6 million paired-end reads and 20 million single-end reads per sample with a length of 150 bp were generated for Col-0- and *pWER*::*FLS2-GFP-*based experiments.

### RNA-seq data analysis

Raw Illumina RNA-seq reads were preprocessed using fastp (v0.19.10)^63^ with the default settings for paired-end (Col-0 experiment) or single-end reads (*pWER*::*FLS2-GFP* experiment). For single-end reads, low-quality sequences from the head (8 bases) and tail (2 bases) were trimmed. High-quality reads were pseudoaligned to the TAIR 10 *Arabidopsis thaliana* transcriptome reference (Ensembl)^64^ using kallisto (v.0.46.1)^65^. On average, 6.7 million paired-end and 18.1 million single-end reads per sample were mapped to the reference *Arabidopsis* transcriptome. After removing low-abundant transcripts that were absent in at least two replicates under each condition, count data were imported using the tximport^66^ package.

Differential expression analyses were performed using the DESeq2 package^67^. First, raw counts were normalized with respect to the library size (rlog function) and log_2_-transformed. We tested for sample batch effects by surrogate variable (SV) analysis using the sva^68^ package. Significant SVs were automatically detected and integrated into the model for differential analyses. PCA (prcomp function) on the basis of whole transcripts was performed and plotted to visualize the cluster and variance of biological replicates under each condition. The abundance of *Arabidopsis* latent virus-1 reads did not correlate with sample variances and therefore removed from downstream analyses. Pairwise comparisons were designed as: (1) flg22 treatment effect only; (2) non-suppressive and suppressive SynCom effect only; (3) flg22 treatment plus SynCom effects; and (4) living versus heat-killed bacteria. Transcripts with fold changes of >1.5 and adjusted *P* values corrected for multiple comparisons (Benjamini–Hochberg method) equal to or below 0.05 were considered to be significant.

The log_2_-scaled counts were normalized by the identified SVs using the limma^69^ package (removeBatchEffect function), and transformed as median-centred *z* score by transcripts (scaled counts, scale function). Then, *z* scores were used to conduct *k*-means clustering for all transcripts. The cluster number (*k* = 10) was determined by the sum of squared error and Akaike information criterion. Next, confirmed transcripts with similar expression patterns were grouped in the same cluster. Differentially expressed transcripts (3,718 in *pWER*::*FLS2-GFP* and 4,450 in Col-0 experiments) and cluster results were visualized using heat maps generated using the ComplexHeatmap^70^ package.

GO enrichment for each cluster using the whole *Arabidopsis* transcriptome as background were performed using the goseq^71^ package with the consideration of transcript length. GO annotations were retrieved from the Gene Ontology Consortium^72,73^ (September 2019). Significantly changed biological process GO terms (adjusted *P* < 0.05) were visualized in dot plots using the clusterProfiler^74^ package. Defence-related genes were extracted on the basis of the GO-term annotation with manual curation. These genes were marked in Supplementary Table 7.

A GO gene network was built by connecting GO terms with shared differentially changed genes (Jaccard similarity > 0.2), such that GO terms holding close function annotations were gathered. Nodes in the network were coloured according to their representation in the *k*-means clustering analysis, while their size corresponded to the number of genes annotated in the corresponding GO term. GO gene networks were visualized in Cytoscape^75^ with a modified configuration from metascape^76^.

DEGs from another two RNA-seq datasets, Teixeira et al.^31^ and Harbort and Hashimoto et al.^13^, were used to confirm genes involved in SynCom response. The RNA-seq analyses pipeline was the same as described above. GO enrichment was conducted on the basis of the common significantly changed genes from this study and published datasets using clusterProfiler package.

### qPCR with reverse transcription

Plants were germinated on half-strength MS plate without sugar in the presence of the indicated SynCom. For chronic response, 1 μM flg22 was added to the agar plate at the beginning of the experiment. For acute response, plants (aged 2 weeks) were submerged with 1 μM flg22 solution in half MS medium for 1 h. Roots were then harvested after 1 h or 24 h. For chitin-related experiments, chitin was dissolved in sterile water at 10 mg ml^−1^ with gentle shaking together with metal beads for a few hours at 4 °C. Resuspended chitin solution was diluted to 1 mg ml^−1^ and autoclaved. The solution was centrifuged to obtain clear supernatant which was subsequently used to flood inoculate plants for 1 h, 6 h or 24 h. Roots from at least five plants (aged 2 weeks) were pooled and total RNA was extracted using the RNeasy Plant Mini Kit (QIAGEN) according to the manufacturer’s instructions. Total RNA (200–500 ng) was treated with DNase, and then processed for first-strand cDNA synthesis using oligo dT primers and superscript II reverse transcriptase (Invitrogen). cDNA was diluted 10 times and 5 µl sample was used as a template for quantitative PCR analysis in a 20 µl reaction mixture supplemented with 1× iQ SYBR Green (Bio-Rad) and 0.2 µM primer each. *UBQ5* (Extended Data Fig. 10a–c) or *SAND* (At2g28390) (Extended Data Fig. 10h) were used for internal normalization. The fold change values were determined using the $2^{{-\Delta\Delta}{c}_{t}}$ method. Statistical analyses were performed on the log-transformed fold changes (Extended Data Fig. 10a–c) and fold changes (Extended Data Fig. 10h) as indicated. A list of the primers used in this study is provided in Supplementary Table 13.

### Statistical analysis

Analyses were performed using the R environment. *t*-tests, Dunn’s Kruskal–Wallis tests, Dunnett’s tests and ANOVA were used to test for statistical significance. Unless otherwise indicated, *P* *<* 0.05 was considered to be significant.

### Reporting Summary

Further information on research design is available in the Nature Research Reporting Summary linked to this article.

## Supplementary information

Reporting Summary

Supplementary Table 1 SynComs used in this study.

Supplementary Tables 2–4 Raw/scale counts and DEGs obtained from RNA-seq data using p*WER*::*FLS2*-*GFP* plants.

Supplementary Tables 5–7 Raw/scale counts of DEGs obtained from RNA-seq data using Col-0 plants.

Supplementary Tables 8–12 Common SynCom-downregulated and general responsive DEGs from multiple studies.

Supplementary Table 13 List of primers used.

## Data Availability

Raw transcriptome and 16S rRNA amplicon sequencing data from this project were deposited at NCBI under the accession number GSE157128. Mass spectrometry data have been deposited to Panorama Public (https://panoramaweb.org/flg22_RGI.url) and the ProteomeExchange (PXD020452). Source data are provided with this paper.

## Source data

Source Data Fig. 1 Statistical source data. Source Data Fig. 4 Statistical source data. Source Data Extended Data Fig. 1 Statistical source data. Source Data Extended Data Fig. 2 Statistical source data. Source Data Extended Data Fig. 2 Unprocessed western blot for Extended Data Fig 2e. Source Data Extended Data Fig. 3 Statistical source data. Source Data Extended Data Fig. 10 Statistical source data.

## Code availability

Scripts to reproduce Figs. 1–4 are available at GitHub (https://github.com/YulongNiu/MPIPZ_microbe-host_homeostasis).
