# Supplementary material for: Coordination of microbe–host homeostasis by crosstalk with plant innate immunity
Source: Nat Plants. 2021 May 24;7(6):814–25. doi: 10.1038/s41477-021-00920-2 (PMC8208891; doi:10.1038/s41477-021-00920-2)
Supplement: Supplementary file 1 — Reporting Summary [file 41477_2021_920_MOESM1_ESM.pdf]

nature research

Corresponding author(s): Ruben Garrido-Oter, Paul Schulze-Lefert

Last updated by author(s): Apr 1, 2021

Reporting Summary

Nature Research wishes to improve the reproducibility of the work that we publish. This form provides structure for consistency and transparency in reporting. For further information on Nature Research policies, see our Editorial Policies and the Editorial Policy Checklist.

Statistics

For all statistical analyses, confirm that the following items are present in the figure legend, table legend, main text, or Methods section.

n/a	Confirmed	
☐	☒	The exact sample size (*n*) for each experimental group/condition, given as a discrete number and unit of measurement
☐	☒	A statement on whether measurements were taken from distinct samples or whether the same sample was measured repeatedly
☐	☒	The statistical test(s) used AND whether they are one- or two-sided<br>*Only common tests should be described solely by name; describe more complex techniques in the Methods section.*
☐	☒	A description of all covariates tested
☐	☒	A description of any assumptions or corrections, such as tests of normality and adjustment for multiple comparisons
☐	☒	A full description of the statistical parameters including central tendency (e.g. means) or other basic estimates (e.g. regression coefficient) AND variation (e.g. standard deviation) or associated estimates of uncertainty (e.g. confidence intervals)
☐	☒	For null hypothesis testing, the test statistic (e.g. F, t, r) with confidence intervals, effect sizes, degrees of freedom and P value noted<br>*Give P values as exact values whenever suitable.*
☒	☐	For Bayesian analysis, information on the choice of priors and Markov chain Monte Carlo settings
☒	☐	For hierarchical and complex designs, identification of the appropriate level for tests and full reporting of outcomes
☒	☐	Estimates of effect sizes (e.g. Cohen's *d*, Pearson's *r*), indicating how they were calculated

Our web collection on statistics for biologists contains articles on many of the points above.

Software and code

Policy information about availability of computer code

Data collection	RNA-Seq raw data was generated by sequncing using the Illumina HiSeq2500 platform. 16S amplicon data was collected using a Illumina Miseq sequencer.

Data analysis	Softwares being used in this study were stated explicitly in the method and summarized below:<br>1. fastp v0.19.10 https://github.com/OpenGene/fastp<br>2. kallisto v0.46.1 https://pachterlab.github.io/kallisto/about<br>3. tximport R package 1.16.1 https://bioconductor.org/packages/release/bioc/html/tximport.html<br>4. DESeq2 R package 1.28.1 https://bioconductor.org/packages/release/bioc/html/DESeq2.html<br>5. sva R package 3.36.0 https://bioconductor.org/packages/release/bioc/html/sva.html<br>6. limma R package 3.44.3 https://bioconductor.org/packages/release/bioc/html/limma.html<br>7. ComplexHeatmap R package 2.4.3 https://bioconductor.org/packages/release/bioc/html/ComplexHeatmap.html<br>8. goseq R package 1.40.0 https://bioconductor.org/packages/release/bioc/html/goseq.html<br>9. clusterProfiler R package 3.16.1 https://bioconductor.org/packages/release/bioc/html/clusterProfiler.html<br>10. Cytoscape 3.8.0 https://cytoscape.org/<br>11. metascape 3.5 https://metascape.org/gp/index.html#/main/step1<br>12. Arabidopsis thaliana gene ontology datasets retrieved on September 2019 http://geneontology.org/<br>13. QIIME http://qiime.org/<br>14. usearch v10 https://www.drive5.com/usearch/<br>15. ggplot2 R package 3.3.2 https://ggplot2.tidyverse.org/<br>16. vegan R package 2.5-6 https://cran.r-project.org/web/packages/vegan<br>17. R language 3.6.0 https://www.r-project.org/<br>18. Custom codes were used to analyze the data and were made available on https://github.com/YulongNiu/MPIPZ_microbe-host_homeostasis.

For manuscripts utilizing custom algorithms or software that are central to the research but not yet described in published literature, software must be made available to editors and reviewers. We strongly encourage code deposition in a community repository (e.g. GitHub). See the Nature Research guidelines for submitting code & software for further information.

Data

Policy information about availability of data

All manuscripts must include a data availability statement. This statement should provide the following information, where applicable:

- Accession codes, unique identifiers, or web links for publicly available datasets
- A list of figures that have associated raw data
- A description of any restrictions on data availability

Raw transcriptome and 16S rRNA amplicon sequencing data from this project were deposited at the Gene Expression Omnibus (GEO) under the accession number GSE157128 (project PRJNA660185). Mass spectrometry data has been deposited to Panorama Public (https://panoramaweb.org/flg22_RGI.url) and ProteomeExchange (PDX020452). We also make all data and scripts available to the reviewers and the editorial office at https://github.com/YulongNiu/MPIPZ_microbe-host_homeostasis. Source data associated with Figs 1,4 and extended data Fig 1-3,10 were available.

Field-specific reporting

Please select the one below that is the best fit for your research. If you are not sure, read the appropriate sections before making your selection.

☒ Life sciences ☐ Behavioural & social sciences ☐ Ecological, evolutionary & environmental sciences

For a reference copy of the document with all sections, see nature.com/documents/nr-reporting-summary-flat.pdf

Life sciences study design

All studies must disclose on these points even when the disclosure is negative.

Sample size	Sample size was determined based on preliminary trials that allow for confident statistical analyses. Sample size was indicated as "n" in the corresponding figures. No statistical methods were used to determine sample size.
Data exclusions	All amplicon sequencing data were used except for biological replicate c of experiment 1 due to potential contamination issue or PCR error.
Replication	Different biological replicates were indicated as different shapes as reported in the corresponding figures.
Randomization	Experiments involving plants grown on agar plates were randomized in the chamber during the course of the experiments.
Blinding	Researchers were not blinded to group allocation, partly due to the obvious phenotype difference associated with a treatment. In general, experiments were independently validated or performed by different researchers to ensure reproducibility of the data.

Reporting for specific materials, systems and methods

We require information from authors about some types of materials, experimental systems and methods used in many studies. Here, indicate whether each material, system or method listed is relevant to your study. If you are not sure if a list item applies to your research, read the appropriate section before selecting a response.

Materials & experimental systems

n/a	Involved in the study
☒	☐ Antibodies
☒	☐ Eukaryotic cell lines
☒	☐ Palaeontology and archaeology
☒	☐ Animals and other organisms
☒	☐ Human research participants
☒	☐ Clinical data
☒	☐ Dual use research of concern

Methods

n/a	Involved in the study
☒	☐ ChIP-seq
☒	☐ Flow cytometry
☒	☐ MRI-based neuroimaging
