# Supplementary figures and images for: Coordination of microbe–host homeostasis by crosstalk with plant innate immunity
Source: Nat Plants. 2021 May 24;7(6):814–25. doi: 10.1038/s41477-021-00920-2 (PMC8208891; doi:10.1038/s41477-021-00920-2)

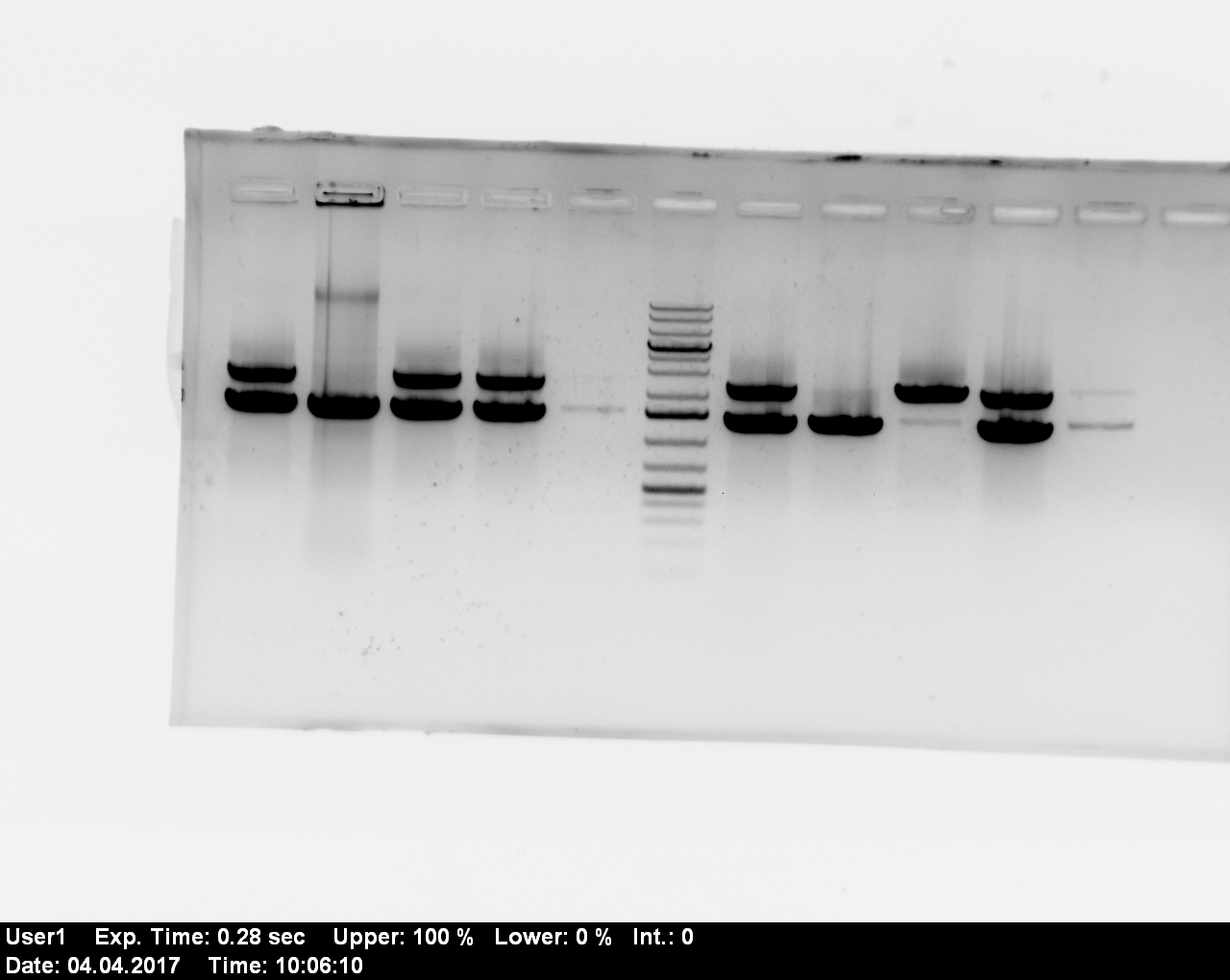

Supplement: Source Data Extended Data Fig. 2 — Unprocessed western blot for Extended Data Fig 2e. [file 41477_2021_920_MOESM11_ESM.tif]
